# Remote thermal detection of exfoliation sheet deformation

**DOI:** 10.1007/s10346-020-01524-1

**Published:** 2020-10-07

**Authors:** Antoine Guerin, Michel Jaboyedoff, Brian D. Collins, Greg M. Stock, Marc-Henri Derron, Antonio Abellán, Battista Matasci

**Affiliations:** 1 Risk Analysis Group, Institute of Earth Sciences, University of Lausanne, 1015 Lausanne, Switzerland; 2 US Geological Survey, Landslide Hazards Program, Moffett Field, CA 94035 USA; 3 US National Park Service, Yosemite National Park, El Portal, CA 95318 USA; 4 School of Earth and Environment, Faculty of Environment, University of Leeds, Leeds, LS2 9JT UK

**Keywords:** Exfoliation, Rockfall source, Terrestrial laser scanning, Infrared thermography, Yosemite Valley

## Abstract

A growing body of research indicates that rock slope failures, particularly from exfoliating cliffs, are promoted by rock deformations induced by daily temperature cycles. Although previous research has described how these deformations occur, full three-dimensional monitoring of both the deformations and the associated temperature changes has not yet been performed. Here we use integrated terrestrial laser scanning (TLS) and infrared thermography (IRT) techniques to monitor daily deformations of two granitic exfoliating cliffs in Yosemite National Park (CA, USA). At one cliff, we employed TLS and IRT in conjunction with in situ instrumentation to confirm previously documented behavior of an exfoliated rock sheet, which experiences daily closing and opening of the exfoliation fracture during rock cooling and heating, respectively, with a few hours delay from the minimum and maximum temperatures. The most deformed portion of the sheet coincides with the area where both the fracture aperture and the temperature variations are greatest. With the general deformation and temperature relations established, we then employed IRT at a second cliff, where we remotely detected and identified 11 exfoliation sheets that displayed those general thermal relations. TLS measurements then subsequently confirmed the deformation patterns of these sheets showing that sheets with larger apertures are more likely to display larger thermal-related deformations. Our high-frequency monitoring shows how coupled TLS and IRT allows for remote detection of thermally induced deformations and, importantly, how IRT could potentially be used on its own to identify partially detached exfoliation sheets capable of large-scale deformation. These results offer a new and efficient approach for investigating potential rockfall sources on exfoliating cliffs.

**Electronic supplementary material:**

The online version of this article (10.1007/s10346-020-01524-1) contains supplementary material, which is available to authorized users.

## Introduction

Rock slope failure leading to rockfall is a dynamic erosional process that controls the evolution of many landscapes and, in particular, steep bedrock formations (Varnes 1978; Hutchinson 1988; Evans and Hungr 1993; Hungr et al. 1999; Rosser et al. 2005; Rabatel et al. 2008; Stock and Uhrhammer 2010; Krautblatter et al. 2012; Janeras et al. 2017). The identification of potential rockfall sources and the detection of deformations prior to failure are crucial for improving rockfall hazard assessment (Terzaghi 1962; Saito 1969; Fukuzono 1985; Zvelebill and Moser 2001; Crosta and Agliardi 2003; Corominas et al. 2005). Over the past 15 years, the rapid development of remote sensing techniques such as ground-based InSAR and LiDAR has dramatically revolutionized the characterization and monitoring of rock mass deformation (Slob and Hack 2004; Rosser et al. 2007; Collins and Sitar 2005, 2008; Oppikofer et al. 2009; Derron et al. 2011; Jaboyedoff et al. 2012; Dehls et al. 2014; Rouyet et al. 2017). High spatio-temporal resolution monitoring of rock cliffs using terrestrial laser scanning (TLS) point clouds (e.g., Abellán et al. 2009, 2010; Rosser et al. 2013; Royán et al. 2014, 2015; Kromer et al. 2015a, 2018; Stock et al. 2018) has shown that it is possible in some cases to detect precursor deformations below centimetric scale and to monitor them over time until failure. In addition to obtaining a more comprehensive rockfall inventory and a more realistic volume-frequency relationship (Barlow et al. 2012; van Veen et al. 2017; Williams et al. 2018, 2019), near real-time TLS surveys offer new opportunities to better anticipate failure (Eitel et al. 2016; Kromer et al. 2015b, 2017). These systems also help to better understand the influence of environmental factors on rockfall triggering (Jaboyedoff and Derron 2005).

A wide range of external environmental factors, including precipitation, freeze-thaw cycles, and thermal effects associated with temperature and insolation, can trigger the failure of unstable rock slopes (Wieczorek and Jäger 1996; Matsuoka and Sakai 1999; Ishikawa et al. 2004; Gunzburger et al. 2005; Frayssines and Hantz 2006; D'Amato et al. 2016; Collins and Stock 2016; Dietze et al. 2017). Regarding daily and seasonal temperature fluctuations in particular, many authors (Vlcko et al. 2009; Gischig et al. 2011; do Amaral Vargas et al. 2013; Bottelin et al. 2013; Collins and Stock 2016; Draebing et al. 2017; Leith et al. 2017; Villarraga et al. 2018; Collins et al. 2018, 2019) have shown that repeated cycles of heating and cooling can generate stresses capable of propagating cracks in both fractured and competent rocks. Other studies conducted on granitic boulders suggest that insolation-induced thermal stresses are of sufficient magnitude to contribute to progressive rock slope degradation by generating elevated tensile stress fields during fracture opening periods (Waragai 1998; McFadden et al. 2005; Eppes et al. 2010, 2016). However, although these studies have continuously measured (sometimes over several years) the deformation and variations in surface temperature, they used only a few local measurement points to monitor the investigated areas. Thus, despite its daily occurrence, no complete thermally induced deformation cycle (i.e., over 24 h) has yet been conducted on the entire surface of a rock outcrop. Further, the known thermal deformation signal of rock outcrops (e.g., Collins and Stock 2016; Eppes et al. 2016) has never been used in conjunction with state-of-the-art remote sensing tools such as TLS to identify potentially unstable rock cliff areas.

Here we present results coupling TLS surveying with infrared thermography (IRT) techniques to further characterize the links between daily thermal changes and rock face deformation. To undertake these studies, we conducted two intraday monitoring campaigns on two granitic cliffs in Yosemite Valley, CA, USA: the Rhombus Wall and El Capitan. Both cliffs are subject to exfoliation processes, wherein partially detached flakes of rock, termed exfoliation sheets, occur along surface parallel fractures (Matthes 1930; Bradley 1963; Bahat et al. 1999; Martel 2006). Due to their partially detached geometry, exfoliation sheets are common sources for rock falls in Yosemite (Wieczorek and Snyder 1999; Stock et al. 2011, 2012; Matasci et al. 2018). Expanding on previous work on the subject of thermally induced deformation and rock fracture of exfoliation sheets by Collins and Stock (2016), we first revisit the site of their experiment on the Rhombus Wall and confirm the diurnal expansion and contraction of a partially detached exfoliation sheet using in situ instrumentation. We then use TLS and IRT methods to characterize the thermal and deformation behavior of the exfoliation sheet in order to identify the 3-D signature of exfoliation sheets subject to diurnal heating and cooling cycles. Finally, using our base knowledge of exfoliation sheet response gleaned from the Rhombus Wall, we apply TLS and IRT methods to remotely detect potentially unstable exfoliation sheets on El Capitan. These efforts suggest a novel method for assessing cliff stability based only on remote monitoring of the cliff’s thermal signature.

## Study sites and geologic setting

Yosemite Valley is a 1-km deep, 14-km long glacier-carved canyon, bounded by steep granitic cliffs cutting the western slope of the Central Sierra Nevada mountain range. Yosemite’s cliffs produce numerous rockfalls every year (up to 80 events documented per year; Stock et al. 2013). Although most rockfalls have been documented to occur due to precipitation-related seepage into rock fractures (Stock et al. 2013), some rockfalls occur due to thermally generated stresses (Collins and Stock 2016) wherein parts of rock cliffs (i.e., especially partially detached exfoliation sheets) expand and contract in response to diurnal variations of temperature. These types of cliffs, including the Rhombus Wall and El Capitan, are the subject of our study as they are well suited for characterization by thermal detection methods.

**Fig. 1:**
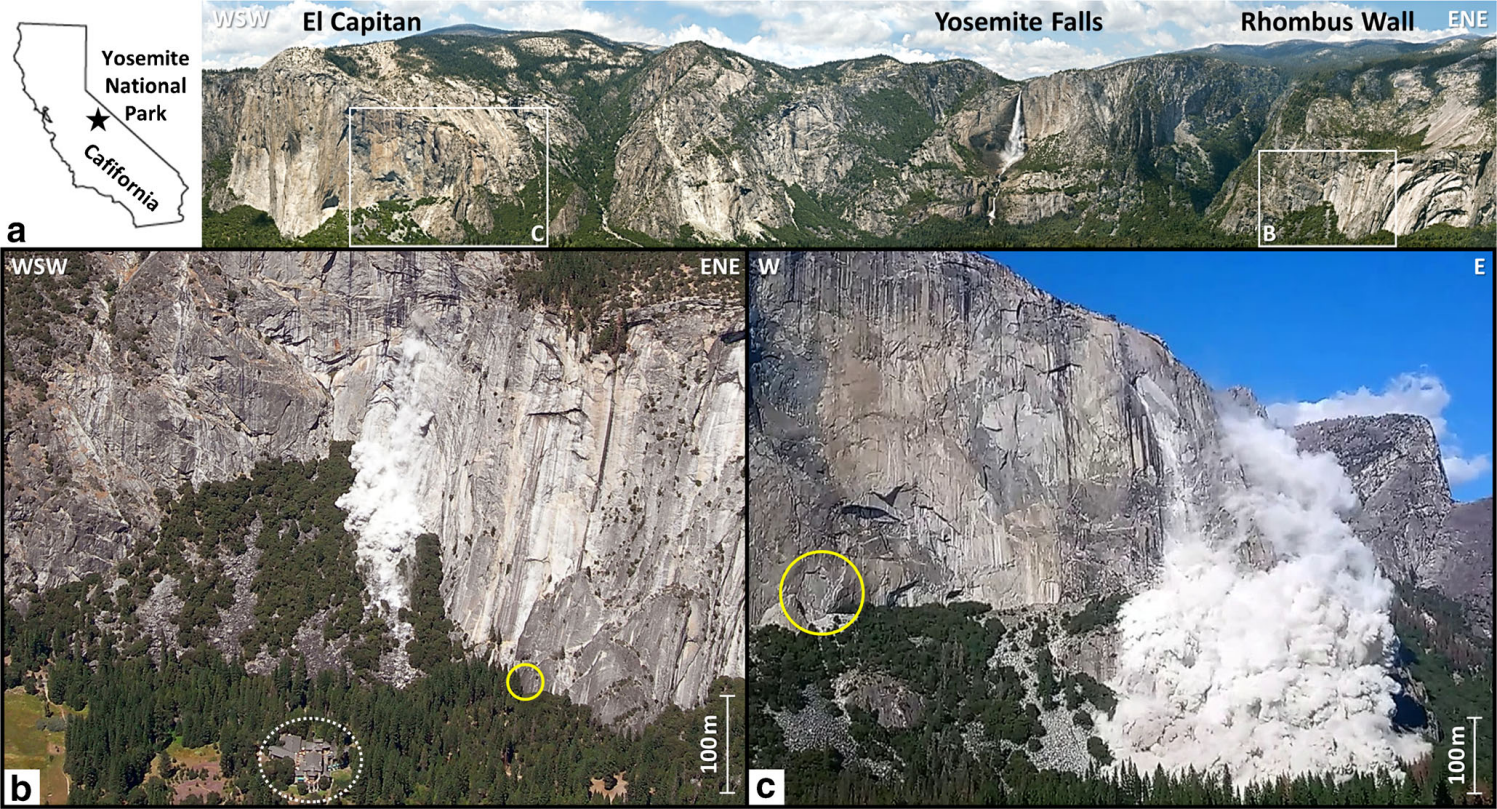
Locations of the two monitored sheeted rock walls in Yosemite Valley. A Gigapixel panorama of the northern wall of Yosemite Valley. Yosemite Falls are visible in the center right of the image. The panorama can be seen at full resolution here: http://gigapan.com/gigapans/49244; photographic credit: Greg Downing (Hypeacuity) and Eric Hanson (BlueplanetVR) (photograph reproduced under an open access license CC BY). B Photograph of a 558 m^3^ rockfall on 26 Aug 2009 rockfall from the south-southwest face of the Rhombus Wall; photographic credit: Nissen Jaffe (photograph reproduced under an open access license CC BY). The yellow circle shows the location of the exfoliation sheet monitored at the base of the Rhombus Wall. The dashed white circle indicates the location of the Ahwahnee Hotel. C Photograph of a 9,811 m^3^ rockfall on 28 September 2017 from the southeast face of El Capitan; photographic credit: Przemek Pawlikowski (photograph reproduced under an open access license CC BY). The yellow circle shows the location of the sheeted wall monitored at the base of El Capitan

**Fig. 2:**
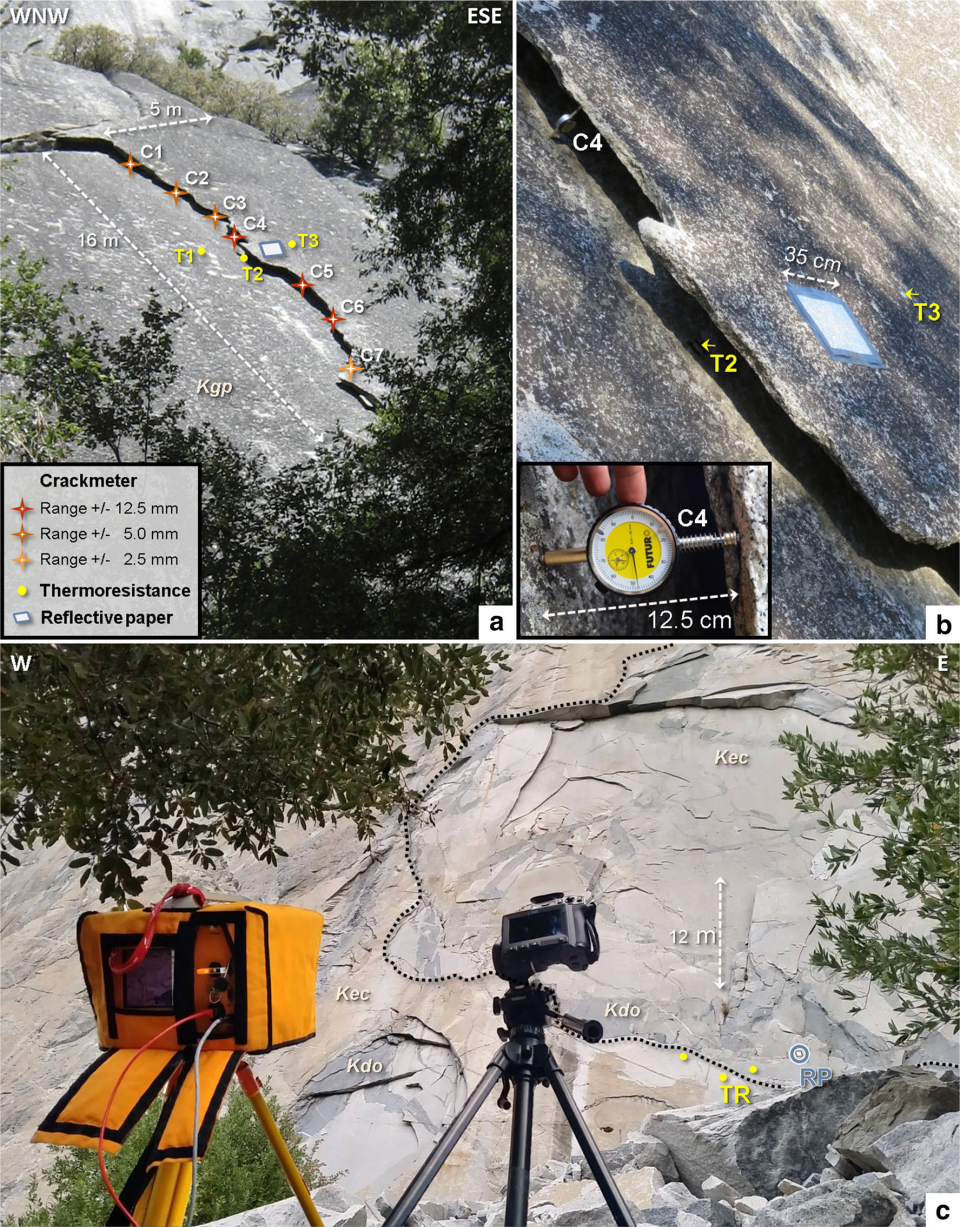
Instrumentation setups at the Rhombus Wall flake and El Capitan. A Side view of the sub-vertical granodiorite exfoliation sheet at the Rhombus Wall flake (time shown is 13 Oct. 2015 at 12:05 PDT). The picture is taken from the position of both laser scanner and thermal camera. C#, crackmeter; T#, thermoresistance sensors; Kgp, Glacier Point granodiorite*.* Red, brown, and orange stars correspond to large, medium, and small size crackmeters, respectively. B Detail view of the area where the reflective paper was fixed on the Rhombus Wall flake (time shown is 13 Oct. 2015 at 16:55 PDT). The shadow on the rock is due to the vegetation surrounding the study site. C Front view of the granitic exfoliating rock wall monitored on El Capitan (time shown is 19 Oct. 2015 at 17:33 PDT). The black dashed line indicates the western boundary of a rockfall scar from 3 October 1976. All overhanging sections correspond to granitic exfoliation sheets. The foreground shows the laser scanner (left) and infrared thermal camera (right). Kec, El Capitan granite; Kdo, dikes of the oceans; RP, reflective paper; TR, thermoresistance sensors

The Rhombus Wall is located north of the Ahwahnee Hotel and east of Yosemite Falls in eastern Yosemite Valley (Fig. 1A, b). This 550-m-tall cliff was affected by a series of rockfalls in 2009–2010 (Stock et al. 2012), with the largest rockfalls on 26 August 2009 (Fig. 1B), leading to temporary evacuation of the hotel. The partially detached exfoliation sheet (19-m tall, 4-m wide, and 10.1-cm thick) that we monitored is located near the base of the Rhombus Wall and, as noted, was previously investigated by Collins and Stock (2016), who instrumented the flake between 2010 and 2013 with crackmeters and temperature sensors. This rock flake is representative of the general geometric features of exfoliation sheets in Yosemite Valley, which are discernibly curved and partially detached near to and sub-parallel to the rock wall surface (Collins and Stock 2016; Martel 2017). Located at the base of the south-southwest face of Rhombus Wall (Fig. 1A, B) at an elevation of 1,255 m, the study area (Fig. 2A) was under the ice cover during the last glacial period (Wahrhaftig et al. 2019). The Rhombus Wall consists of Glacier Point granodiorite (Calkins et al. 1985; Peck 2002) that belongs to the Late Cretaceous Tuolumne Intrusive Suite and was emplaced from 95 to 85 Ma (Coleman et al. 2004; Memeti et al. 2010). The Tuolumne Intrusive Suite intruded from the east into the intrusive suite of Yosemite Valley that includes the El Capitan granite (Bateman 1992).

With its 900-m-tall cliffs, El Capitan in western Yosemite Valley is one of the most iconic granite formations in Yosemite (Fig. 1A). El Capitan has many prominent exfoliation sheets and has experienced many exfoliation-type rockfalls (Stock et al. 2013). The easternmost part of its southeast face was affected by a rockfall sequence during the autumn of 2017 whose largest volume (8,611 m^3^) collapsed on 28 September 2017 (Stock et al. 2018; Guerin et al. 2020). Unlike the Rhombus Wall, El Capitan consists of eight different rock units, including three types of granite and two types of dikes (Putnam et al. 2015). The El Capitan granite (106.1 ± 0.5 Ma) is the primary unit exposed on the southeast face (Ratajeski et al. 2001; Putnam et al. 2015). The El Capitan granite is regularly cut by a series of dioritic to granodioritic dikes (dikes of the oceans; Putnam et al. 2015). Only these two rock types outcrop in the sub-vertical rock wall we monitored. Our 50-m-tall by 40-m-wide study area on El Capitan is located at the base of the southeast face of El Capitan (Fig. 1C), at an elevation of 1,475 m (i.e., above the trimline of the last glaciation; Wahrhaftig et al. 2019), and corresponds to the location of a rockfall scar from 3 October 1976 (1,864 m^3^; Guerin et al. 2020).

## Data collection

### Crackmeter measurements

To measure and confirm the deformation behavior of the Rhombus Wall exfoliation sheet, we installed seven temporary crackmeters (standard analog comparators with springs specially adapted for this study) along the edge of the Rhombus Wall flake*. These were installed* between the rock sheet itself and the adjacent cliff which we considered as stable. The data from these sensors also allowed us to calibrate and assess the accuracy of our TLS measurements. Depending on the crack aperture, which varies between 8 and 13 cm, three sizes of crackmeter were used. The largest crackmeters (Fig. 2A; C4, C5, C6) were placed in the central part of the rock sheet edge where the crack aperture is maximum (Fig. 2B); these sensors can measure a range of deformation up to of ± 12.5 mm with an accuracy of ± 0.01 mm. Mid-sized crackmeters (maximum range, ± 5.0 mm; accuracy, ± 0.01 mm) are positioned in the upper part of the rock sheet (Fig. 2A; C1, C2, C3), while the smallest crackmeter (maximum range, ± 2.5 mm; accuracy, ± 0.01 mm) is placed in the lower part of the flake (Fig. 2A; C7). We measured the crackmeter manually at 2-h intervals beginning on 13 October 2015 at 20:23 Pacific daylight time (PDT) and ending on 14 October 2015 at 18:23 PDT. Due to the dangerousness of the monitored rock wall at El Capitan and the lack of accessible open fractures, no crackmeter could be installed in this cliff.

### TLS and IRT acquisitions

To capture the 3-D intraday deformation of exfoliation sheets, we collect and compare time series of TLS scan data of both study areas (Rhombus Wall and El Capitan; Fig. 1). We collected all TLS point clouds using an Optech ILRIS-LR laser scanner with a manufacturer-specified accuracy of 7 mm for a single point at a range of 100 m (Teledyne Optech 2020). For our study, all cliff areas are within this range (35 m for the Rhombus Wall flake, Fig. 2A, and 72 m for the El Capitan study area, Fig. 2C). We measured rock surface temperature variations using a FLIR T-660 infrared thermal camera with a measurable temperature range between −40 °C to + 2,000 °C, an accuracy of ± 1 °C, and a thermal resolution less than 0.2 °C (FLIR 2014). The FLIR T-660 camera has an infrared resolution of 640 × 480 pixels and is equipped with a 5-million-pixel digital RGB camera. The IRT camera possesses a field of view of 25° × 19°, a focal length of 25 mm, and an infrared spectral range of 7.5–14 μm (FLIR 2014). At the above-indicated distances, the pixel resolution (IRT camera) is 2.4 cm for the Rhombus Wall flake and 5.0 cm for the El Capitan study area.

Our TLS/IRT survey at the Rhombus Wall began on 13 October 2015 at 20:00 PDT and ended on 14 October 2015 at 20:00 PDT, thereby capturing a full 24-h cycle. We collected TLS scans (8 million points; 4.5 mm point spacing on average, i.e., ~ 50,000 pts/m^2^) every hour and thermal photographs (640 × 480 pixels) every 20 min (73 thermograms in total). Our survey at El Capitan started on 19 October 2015 at 17:30 PDT and ended on 20 October 2015 at 01:30 PDT, for a total of 8 h. Here, the aim was to be able to confirm some results highlighted for the Rhombus Wall flake, and not to characterize another full cycle of thermally induced deformations. Furthermore, no crackmeter or thermoresistance (Fig. 2C) could be fixed in open fractures. We conducted TLS acquisitions (27 million points; 8.5 mm point spacing on average, i.e., ~ 14,000 pts/m^2^) every hour and thermal photographs every 20 min. A total of 50 thermograms were collected at El Capitan because two IRT images were needed to cover this study area.

### Thermoresistance and thermal sensor measurements

We used five thermal sensors to calibrate the apparent temperatures measured by the IRT camera: three thermoresistance sensors (PT100), a reflective paper, and a thermohydrometer. At the Rhombus Wall flake, we use the three thermoresistance sensors (fixed with an electrician’s insulating tape patch) to obtain temperature values on the reference surfaces, inside the fracture (in the shadow of the rock sheet but on the adjacent cliff surface) and on the rock sheet surface (Fig. 2A, B). For the El Capitan study site, thermoresistance sensors are fixed at the base of the cliff on an outcropping of El Capitan granite, the most widely exposed rock type on the cliff (Fig. 2C). The reference temperatures given by the thermoresistance sensors also enabled us to determine the emissivity of the Glacier Point granodiorite at Rhombus Wall (0.95) and the El Capitan granite at the El Capitan cliff (0.81). We recorded thermoresistance measurements manually every 2 h for the Rhombus Wall flake and in 1-hour intervals at El Capitan. PT100 sensors have a measurable temperature range between 0 °C and + 150 °C with an accuracy of ± 0.1 °C (class 1/3 DIN; Heraeus 2020).

For calibration of the two IRT surveys, we fix the reflective paper (crumpled then uncrumpled aluminum foil) near the thermoresistance sensors (Fig. 2). This allows for measuring the reflected apparent temperature (see Supplementary Tables 1 and 2) at the rock surface on each thermogram. In these tables, the average of the temperatures displayed by the crumpled and then uncrumpled reflective paper is reported. During each IRT acquisition (every 20 min), we measure the ambient air temperature and relative humidity readings (see Supplementary Tables 1 and 2) using a DIGIHUM2 pocketsize digital thermohydrometer. This device measures the ambient air temperature from −20 °C to + 60 °C with an accuracy of ± 1 °C and the relative humidity from 20 to 95% with an accuracy of ± 1% between 30 and 80% (± 5% outside this range; Littoclime 2014).

## Data processing

### TLS data processing

We processed TLS point clouds using four main stages:( 1) cleaning of raw scans by removing outlier points and vegetation; (2) alignment of point clouds into a local coordinate system using only the reference areas considered as stable; (3) generation of the reference mesh and point-to-surface registration; and (4) calculation of distances between the reference mesh and the successive scans. Step 1 consists of manually selecting and removing vegetation (a few shrubs in our case) and outliers on raw TLS data. Step 2 begins with a coarse point-to-point registration that requires manually selecting several pairs of homologous points in the reference scan and the successive scans. Here, the reference point clouds are the first scans that were acquired. The coarse alignment was then followed by a fine registration using the iterative closest point (ICP) algorithms (Besl and McKay 1992; Chen and Medioni 1992) implemented in CloudCompare software (Girardeau-Montaut 2015).

To optimize the accuracy of the registration methods, we only apply the ICP algorithm to manually selected reference areas considered as stable (see example in Fig. 4); the resulting 4 × 4 roto-translation matrices were then applied to the rest of the point clouds. We generated the triangular reference meshes for both study areas using the Poisson surface reconstruction algorithm (Kazhdan et al. 2006) available in CloudCompare software. This method allows generating a denoised surface while preserving the main curvatures of the 3-D model. It involves (1) a regular sub-sampling (1 point out of 3 for the Rhombus Wall flake; 1 point out of 12 for El Capitan); (2) conversion of the oriented points (normals) into a continuous vector field in 3-D; and (3) determination of a scalar function whose gradients are best suited to the vector field; and (4) extraction of a triangular isosurface. As the accuracy of alignment is one of the leading error sources affecting change detection between two point clouds (Teza et al. 2007; Williams et al. 2018), we applied a point-to-surface ICP registration (Zhang 1994) to reference areas considered as stable using the reference meshes. At the end of step 3, the point-to-surface standard deviations (confidence interval given by *±* 2σ) in the reference areas were ± 1.43 mm for the Rhombus Wall flake and ± 1.29 mm for the *El Capitan study area.* We calculated the raw point-to-mesh distances (step 4) along the normal mean vector associated with each study site. For our surveys, this approximation was acceptable since the investigated topographic surfaces are very flat and present *only slight variations in spatial orientation*. We computed normal mean vectors from the vertices of the reference meshes using between 15 and 30 neighboring points.

**Fig. 3:**
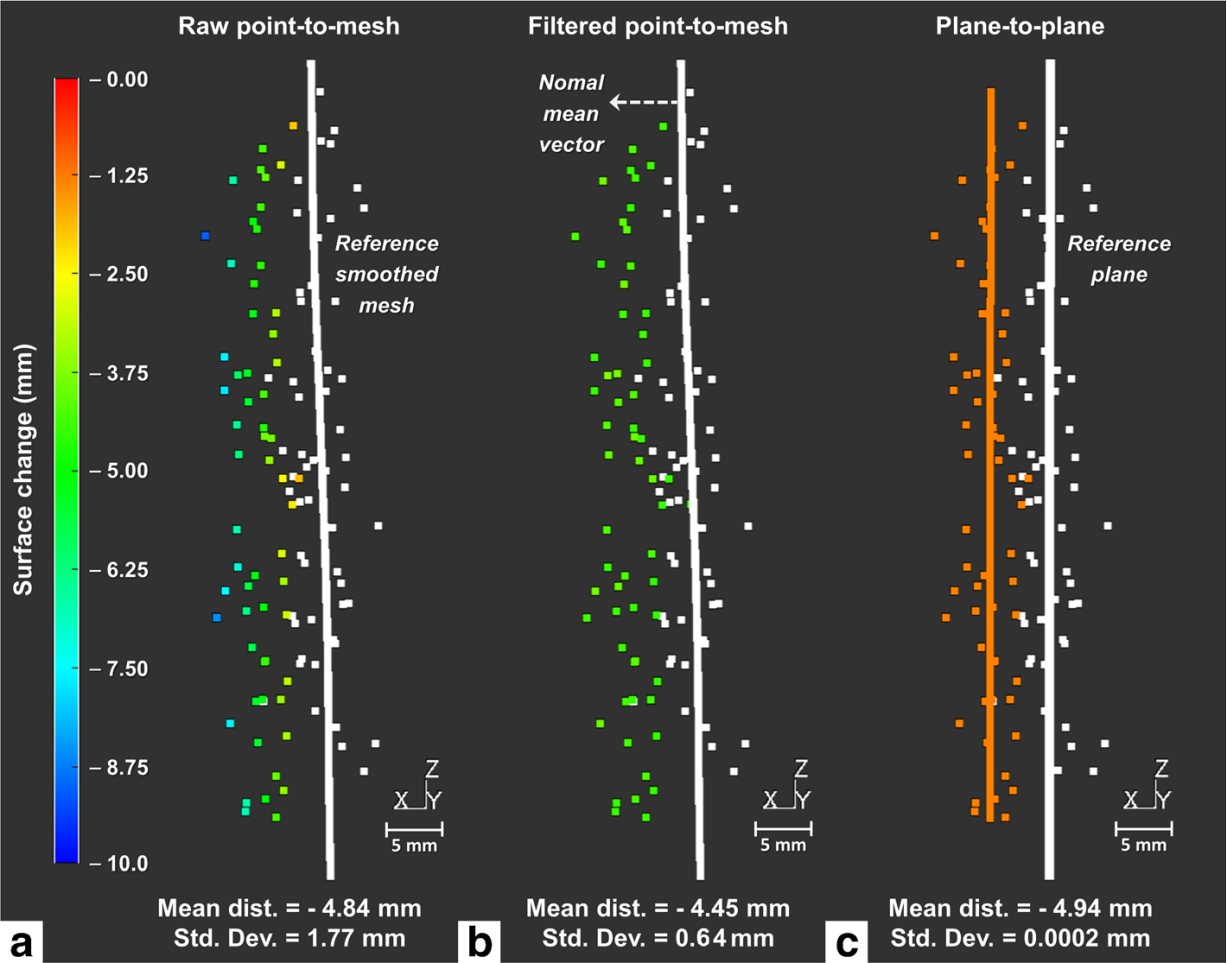
Illustrations of the filtered point-to-mesh and plane-to-plane calculation methods used to denoise the results of the raw point-to-mesh distance calculation. Example for the Rhombus Wall flake between 20:00 and 09:00 PDT in the crackmeter C5 area. For ease of comparison with the labelled deformation values, the deformation measured in C5 at 08:23 PDT is −5.33 mm ± 0.01 mm (Fig. 5C). Standard deviation values correspond to a 95% confidence interval (2σ). **a** Raw point-to-mesh distances between the smoothed reference mesh (in white) and the points of the compared cloud (colorized by distance). Raw point-to-mesh distances are defined as the distances along the normal mean vector (e.g., dashed arrow in **b**). **b** Filtered point-to-mesh distances using the sliding average method. **c** Plane-to-plane distances between the reference plane (in white) and the plane fitted on the points of the compared cloud (in orange). Plane-to-plane distances are defined as the orthogonal distances between the two planes

Given the close range (< 100 m) and high point density of the TLS data (< 1 cm), all the 3-D models are very noisy (Fig. 3). Thus, the raw point-to-mesh distances are subject to a high standard deviation. For example, in the area of crackmeter C5, the 2σ standard deviation is *±* 1.77 mm between 20:00 and 09:00 PDT (Fig. 3a) compared with measurements made with the crackmeter. To overcome this limitation, we tested two noise reduction spatial methods: a semi-automatic filtering algorithm (*sliding average method*; Abellán et al. 2009) which applies to the entire point cloud and a local method of fitting planes. The sliding average method allows denoising the raw point-to-mesh distances using nearest neighbor averaging; here, we used 1000 neighbors for the averaging. This method provides better resolved deformation patterns and significantly reduces the standard deviation (2σ = *±* 0.64 mm; Fig. 3b) of the point-to-mesh distances. However, the filtered point-to-mesh distances are accompanied by an overall underestimation of the deformation values, compared with the values from crackmeter measurements (Fig. 5; see also Supplementary Fig. 1). To limit this change detection bias, we fitted planes oriented with the same normal vector over areas of approximately 1 dm^2^ (~ 500 pts), on both the reference point cloud and the successive point clouds (Fig. 3c). For both TLS surveys, the fitting plane method was applied to a dozen sectors distributed along the edges of the rock sheets (i.e., in the crackmeter areas for the Rhombus Wall flake) and in the reference areas. Plane-to-plane distances are characterized by a near-zero standard deviation (2σ = *±* 0.0002 mm; Fig. 3c) and are only affected by the fluctuating value of the plane-to-plane registration error in the reference areas, namely ± 0.73 mm (2σ) for the Rhombus Wall flake and ± 0.55 mm (2σ) for El Capitan. This systematic residual error can come from the variable atmospheric conditions (ambient air temperature and relative humidity) measured during the two surveys (see Supplementary Tables 1 and 2) and/or circulation of hot air (Jaboyedoff et al. 2012) induced by the nocturnal restitution of the heat stored by the rock during the day.

### IRT data processing

Infrared radiation measured by an IRT camera is composed of direct and indirect radiation (as reflected by the target object), respectively, coming from the camera environment and the target object environment (Shannon et al. 2005; Prendes-Gero et al. 2013; FLIR 2014; Usamentiaga et al. 2014). To correct the influence of these different sources of radiation on the measurement of apparent temperatures, five calibration parameters must be specified: (1) the target object’s emissivity; (2) the reflected apparent temperature; (3) the ambient air temperature; (4) the relative humidity; and (5) the distance between the target object and the IRT camera. Depending on the acquisition strategy, these calibration parameters can either be set directly at the time of acquisition or modified during post-processing. This results in a calibrated thermogram image.

To correct the shifts of a few pixels due to the IRT camera handling (switching on/off, focusing, temperature measurement and acquisition), we applied the *intensity-based automatic image registration* algorithm (MATLAB 2020) to all the thermograms following calibration. This algorithm includes an iterative process, which seeks to spatially optimize the pixels of similar intensity using affine transformations (to preserve collinearity). For this study, we exported all IRT images belonging to the same monitoring campaign into CSV format with identical temperature scale and then applied only roto-translations. Additionally, to avoid abrupt intensity (temperature) changes between two thermograms, each image at *t*_*i + 1*_ was aligned on the previous image at *t*_*i*_; a sub-pixel accuracy (< 20 mm at 35 m distance) characterizes this registration process.

### Rhombus Wall flake animations

We generated four video animations showing the daily evolution of the deformation pattern and surface temperatures of the Rhombus Wall flake following the data processing. These videos are made from the results (as PNG pictures) of the 24 TLS comparisons (see Supplemental Movies 1 and 2), the 12 manual measurements carried out for each crackmeter (see Supplementary Movie 3), and the 73 aligned IRT images (see Supplementary Movie 4). A surface change scale between −5 mm and +1 mm and a temperature scale between +20 °C and +40 °C were, respectively, fixed on 3DReshaper software and MATLAB before generating the videos. These animations provide useful tools for visualizing the coupled thermo-deformation behavior of the cliffs.

(MP4 5599 kb)(MP4 712 kb)(MP4 468 kb)(MP4 18445 kb)

## Results and discussion

### Confirmation of 3-D daily deformation behavior of Rhombus Wall flake

**Fig. 4:**
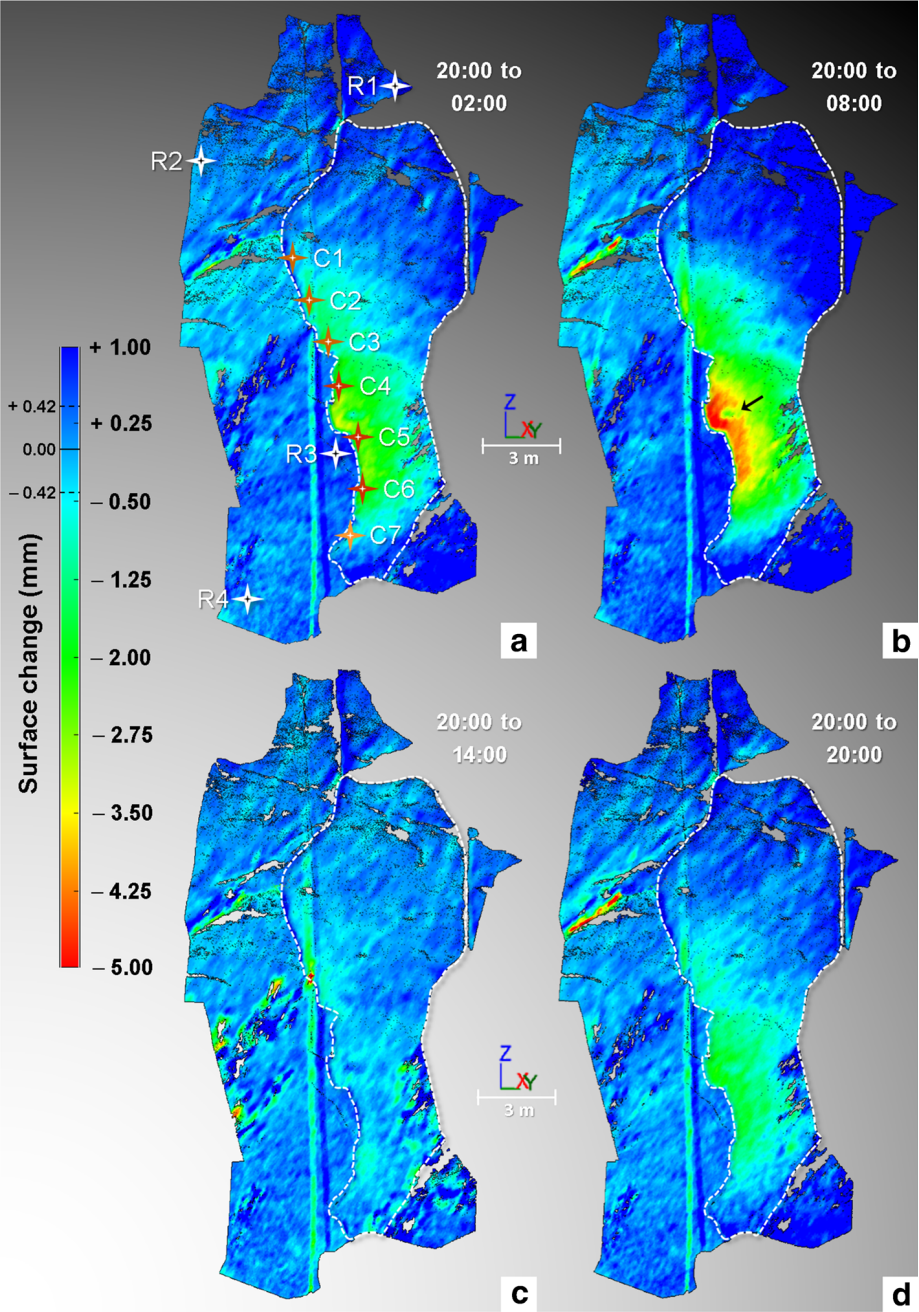
Results of the TLS monitoring conducted on 13–14 Oct. 2015 on the Rhombus Wall flake. (A), (B), (C), and (D) Filtered point-to-mesh differences measured from the first TLS point cloud acquired on 13 Oct. 2015 at 20:00 PDT (the full cycle of TLS comparisons is available in Supplementary Movie 1). The white dashed line delimits the extent of the rock sheet; all other areas were considered as stable (i.e., reference areas). A shows the positions of the seven crackmeters (C1 through C7; Fig. 2A) installed in the fracture, and those of the four areas selected to quantify the surface changes in the reference parts (labelled R1 through R4). Each comparison is characterized by a registration error in the reference areas of ± 0.42 mm (2σ). Negative surface changes indicate an inward deformation pattern that results in a narrowing of the crack width during overnight cooling. Positive surface changes greater than + 0.42 mm (dark blue color) are due to border effects (incidence angle errors). The black arrow indicates the position of the reflective paper; it slightly peeled off during the survey, which explains why the orange-red deformation pattern (B) is discontinuous at this location

**Fig. 5:**
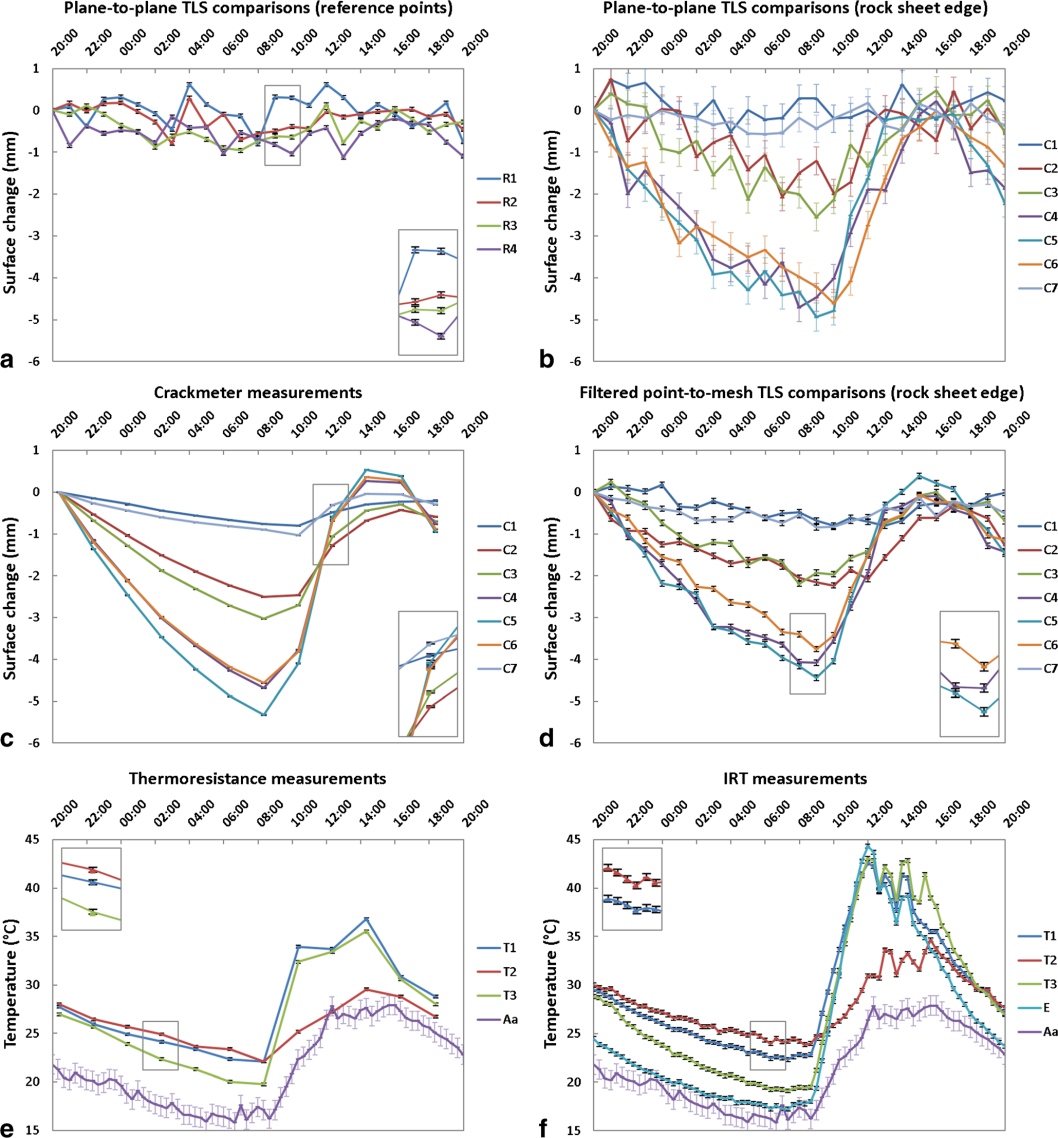
Daily deformation and temperature data of the Rhombus Wall flake on 13–14 Oct. 2015. (A) Deformations measured in the stable reference areas. R# labels represent the TLS data from the four white crosses of Fig. 4 (A). (B), (C), and (D) Deformations measured along the rock sheet edge (see also Supplementary Movies 2 and 3 for deformation cycle animations). C# labels represent either the TLS data from the seven colored crosses of Figs. 2A and 4A (B, D; data measured every hour since 20:00 PDT) or the data from the crackmeters (C; data recorded every two hours since 20:24 PDT). (E), (F) Measured thermal cycles. T# labels represent either the data from the three thermoresistance sensors (E; data recorded every two hours since 20:24 PDT) or the IRT data from the three yellow points of Figs. 2A and 7A (F; data measured every 20 minutes since 20:00 PDT). A and E labels represent the ambient air temperature data and the IRT data from the rock sheet edge (location in Fig. 7A), respectively. Error bars reflect the standard error of the mean of surface changes (A, B, C, D) and the standard error of the mean of temperature values (E, F). Insets show enlarged sections for detail

Our measurements using manual crackmeter readings confirm the daily cyclic thermal deformation response of the Rhombus Wall flake previously investigated by Collins and Stock (2016). Namely, the exfoliation sheet moves outward (away from the rock cliff; positive values in Fig. 4) during morning and early afternoon warming and then moved inward (toward the rock cliff; negative values in Fig. 4) during late afternoon and nocturnal cooling. However, TLS imaging made from the filtered comparisons (Fig. 4; see also Supplementary Movie 1) further allows for investigation of other parts of the sheet that did not have direct crackmeter measurements. Overall, daily inward and outward deformations affecting the Rhombus Wall flake are spatially of the same order of magnitude. However, the largest deformations are located along the rock sheet edge and more specifically in the area of crackmeters C4 through C6 (Figs. 4 and 5; see also Supplementary Movies 2 and 3), where the fracture aperture is the largest (Fig. 2B). During our monitoring, the contraction and expansion peaks (both measured at C5) reach their minimum (−5.33 mm) and maximum (+ 0.53 mm, i.e. a cumulative value of +5.86 mm) values between 08:21 and 09:00 PDT and between 14:21 and 15:00 PDT, respectively (Fig. 5C, D). Thus, the thermomechanical response of the rock sheet is shifted in time with a delay of 01:20 ± 00:20 PDT from the moment where rock surface temperatures are minimum (between 07:00 and 07:40 PDT; Fig. 5F) and a delay of 02:40 ± 00:20 PDT when temperature is maximum (between 11:40 and 12:20 PDT; Fig. 5F).

The comparison of the two noise reduction methods applied to the TLS data with the data from crackmeters shows that, overall, the plane-to-plane method provides deformation values closer to those of crackmeters (Figs. 3 and 5; see also Supplementary Fig. 2). This is because this method has the advantage of locally minimizing the measurement noise and thus is solely influenced by the average registration error of ± 0.73 mm that characterizes the alignment accuracy in the reference areas assumed as stable (Fig. 5A). Still, the plane-to-plane method only provides local deformation values, while the filtering method allows visualizing the deformation pattern at the scale of the entire outcrop. In addition, it is worth mentioning that the surface deformations measured with crackmeters and TLS do not always represent the same phenomenon because the TLS data are subjected to solar radiation (outer face of the rock sheet), whereas those given by crackmeters (inner face) are not. This difference generates a diurnal differential expansion between the outer and inner faces of the flake. Since the TLS measurements capture the expansion of the crack and the expansion of the rock during daytime hours of the day, the deformation values derived from TLS should show larger values than crackmeters during this period. Unfortunately, due to the arrival of a cloudy sky from midday, this effect is not visible for the deformations measured in the afternoon (Fig. 5B, C). By contrast, some higher deformation values (whose shifts with crackmeter data are greater than the average registration error) have been highlighted in the late morning with the plane-to-plane method. The most representative values were measured in the area of crackmeters C4 and C5 with respective differences of + 0.85 mm (−2.92 mm (TLS) versus −3.77 mm (crackmeter); Fig. 5B, C) and + 1.61 mm (−2.19 mm (TLS) versus − 4.10 mm (crackmeter); Fig. 5B, C) between 10:22 and 11:00 PDT.

### 3-D diurnal thermal imaging of Rhombus Wall flake

**Fig. 6.**
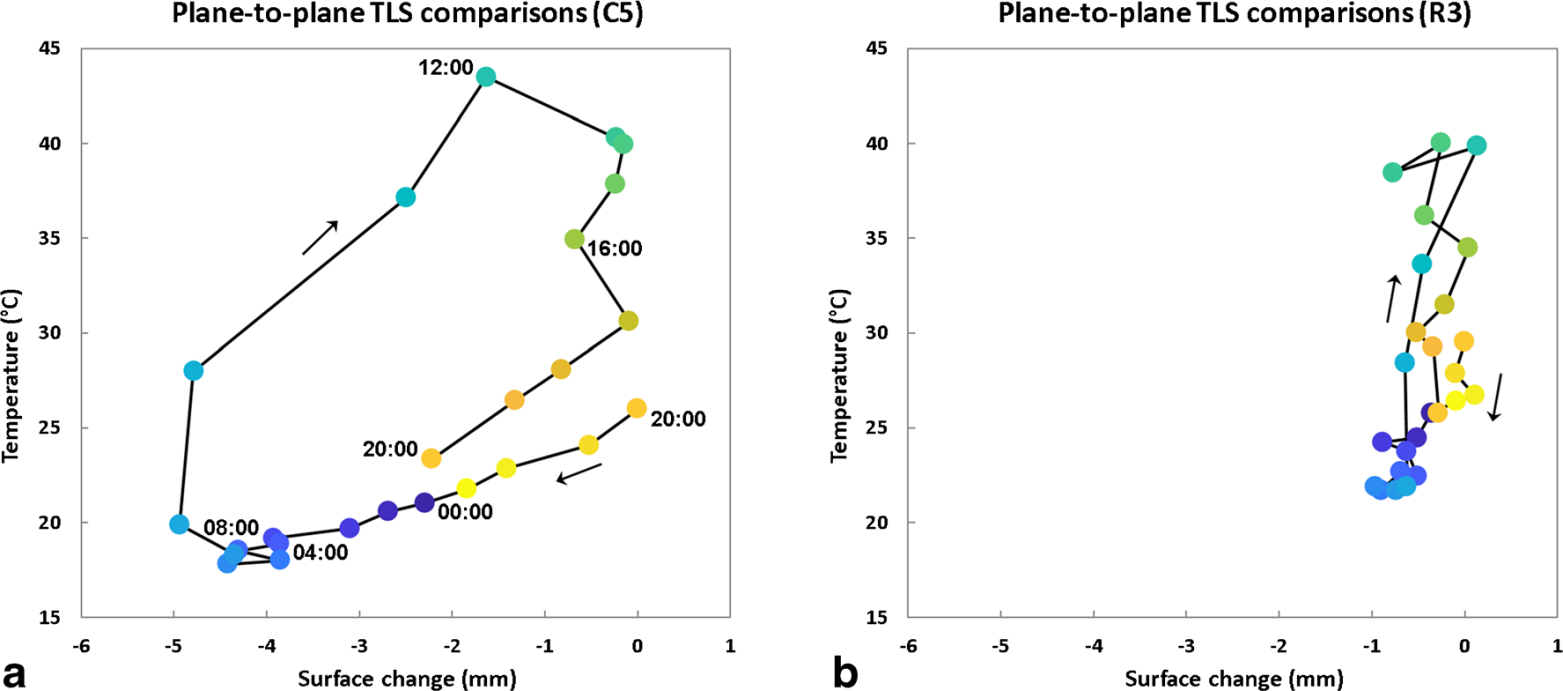
Daily hysteresis loop of thermally induced deformations measured over the Rhombus Wall flake (see also Collins and Stock (2016) for an evolution over several days). Crack closure-aperture cycle (rock sheet deformation) measured at (**a**) the location of crackmeter C5 and (**b**) outside the rock sheet at the location of the reference point R3 (see Fig. 4A for locations). The surface change data come from the plane-to-plane TLS comparisons (Fig. 5B) and the temperature data come from the IRT measurements (Fig. 5F). All times are in PDT (clockwise direction) and the colors associated with them have been chosen arbitrarily to make it easier to follow the cycle over time (in particular in Panel B)

Our 3-D monitoring of an entire cliff area using TLS and IRT methods allowed us to identify the thermal characteristics specific to cyclically deforming exfoliation sheets. The comparison of the hysteresis loops (Fig. 6) determined at the rock sheet edge and in the reference areas considered as stable indicates that the amplitude of daily variations within the Rhombus Wall flake area is higher not only for deformations but also for temperatures. This finding is borne out throughout the day as the rock sheet surface exhibits colder nocturnal temperatures and then warmer diurnal temperatures than the reference areas (Figs. 5F and 6; see also Supplementary Movie 4). Still, it should be specified that the temperature values calibrated on the thermal camera are overall higher compared with the thermoresistance measurements (Fig. 5E, F; see also Supplementary Fig. 2). More specifically, the average deviation in absolute value between measured (thermoresistance sensors) and calibrated (IRT) temperatures is 0.9 °C for nighttime temperatures (19:00–09:00 PDT), whereas it is 3.9 °C for daytime temperatures (see Supplementary Fig. 2). This difference is most probably due to direct and indirect solar radiation that interferes with the measurement of the apparent reflected temperature on the reflective paper, inducing calibration errors. In any case, by investigating the detailed IRT images further and throughout a 24-h cycle, we are able to elucidate additional characteristics that can potentially be used to identify partially detached exfoliation sheets in general.

**Fig. 7:**
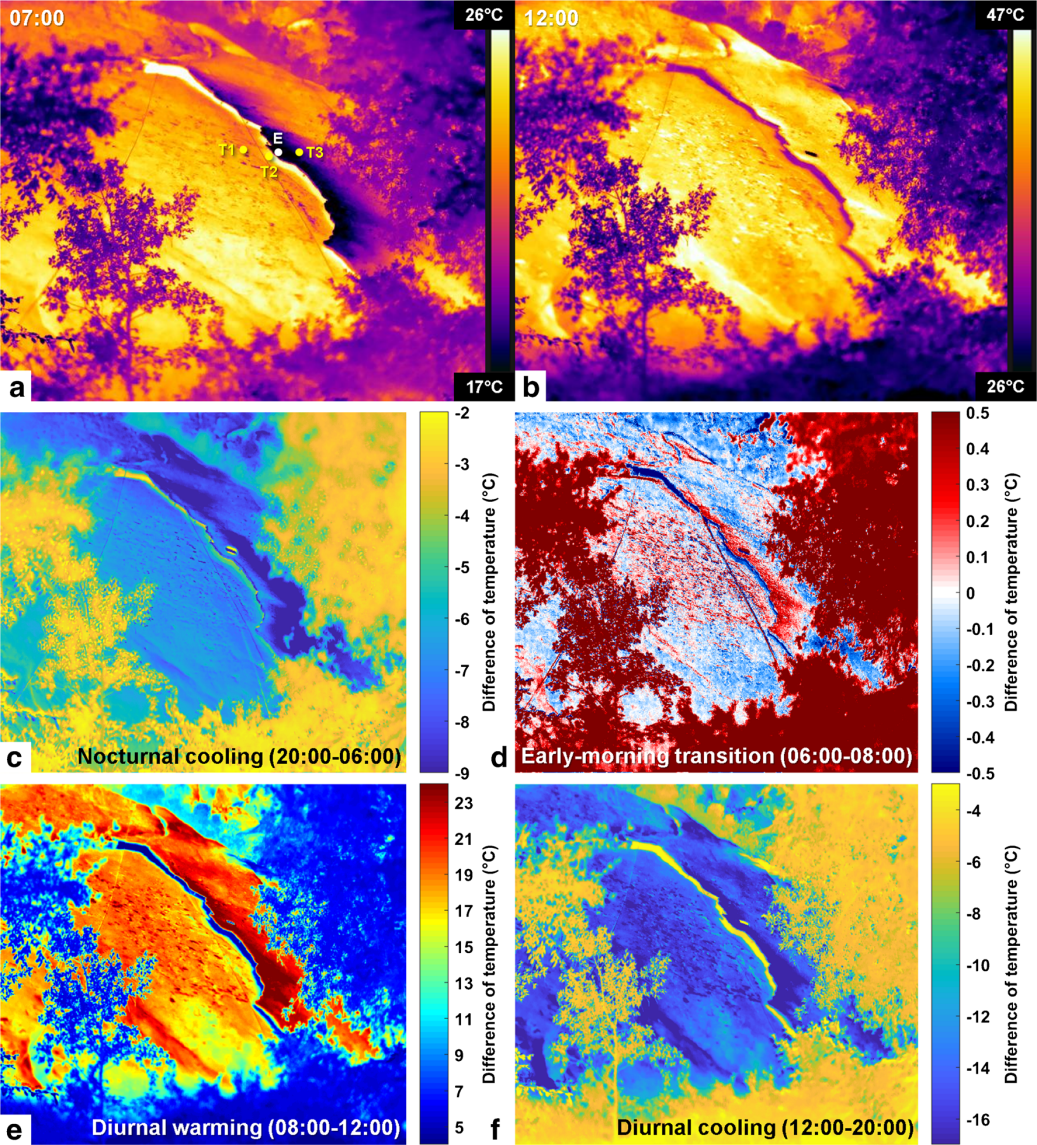
Results of the IRT monitoring on the Rhombus Wall flake. (A), (B) Thermal signatures of RWF on 14 Oct. 2015 at 07:00 and 20:00 PDT, respectively (all thermograms are visible in Supplementary Movie 4). A indicates the positions (in yellow) of the three thermoresistance sensors (T1 through T3) fixed to the rock; the white point shows the position chosen to measure the temperature of the rock sheet edge (labelled E) on IRT images (see Fig. 5F). (C), (D), (E), (F) Temperature differences measured between 20:00 and 06:00 PDT, 06:00 and 07:00 PDT, 07:00 and 12:00 PDT, and 12:00 and 20:00 PDT, respectively. Although the rock sheet edge is the coldest portion between 20:00 and 06:00 PDT, (C) shows that the most significant nocturnal temperature variations occur on the central portion of the rock sheet. By contrast, after a transition period during which the rock sheet edge begins to warm up while the other portions of the rock sheet continue to cool (D), the most significant diurnal temperature variations occur along the rock sheet edge (E, F)

The comparison with IRT images (Fig. 7) shows that the most significant temperature variations are usually located along the rock sheet edge (Fig. 7C, D, E, F). These are coincident with the maximum deformation areas (Fig. 4B). Only the nocturnal cooling period shows a different behavior since, although the rock sheet edge is the coldest portion between 20:00 and 06:00 PDT (Fig 5F), this is the central part of the Rhombus Wall flake (located east of the reflective paper) that undergoes the most significant nocturnal cooling (ΔT3_IRT-night_ of −9.3 ± 1.1 °C) (Figs 5F and 7c). During daytime hours, the rock sheet displays a thermal transition period in the early morning (06:00–08:00 PDT) when the central part continues to cool, while the edge begins to warm up (Fig. 7D), and then follows a cycle of heating and cooling where the rock sheet edge undergoes the most significant diurnal temperature variations (Fig. 7E, F) with a ΔE_IRT-morning_ of +26.7 ± 6.5 °C between 06:00 and 12:00 PDT and a ΔE_IRT-afternoon_ of −20.6 ± 6.5 °C between 12:00 and 20:00 PDT (Fig. 5F).

The daily thermal pattern detected for the Rhombus Wall flake is reminiscent of that observed for horizontal rectangular fin arrays under natural convection (Goshayeshi and Ampofo 2009; Dhanawade Hanamant et al. 2013). In the field of heat transfer, fins are surfaces that extend from an object (mostly comb-shaped) to increase the rate of heat transfer to or from the environment by increasing convection (Lienhard IV and Lienhard V 2019). As demonstrated by Guerin et al. (2019), the colder edge of the rock sheet is explained by an air circulation that envelops and cools the detached portion of the cliff. By natural convection and/or forced in case of strong wind, the Rhombus Wall flake behaves like a heat sink that generates thermal gradients across and along the rock sheet. Research on the effects of heating on partially detached rock slabs (Collins and Stock 2016; Collins et al. 2018, 2019) has shown that thermal gradients across the thickness of exfoliation sheets likely cause differential stresses between the hot side (outer), subject to tension, and the cooler side (inner), subject to compression. Here, the combination of TLS with IRT shows that the same phenomenon occurs laterally over the rock sheet surface, resulting in an asymmetrical lateral buckling mechanism whose amplitude varies with the crack aperture, and probably also with the thickness of the rock sheet.

### Remote detection of exfoliation sheets on El Capitan from thermally induced deformations

**Fig. 8:**
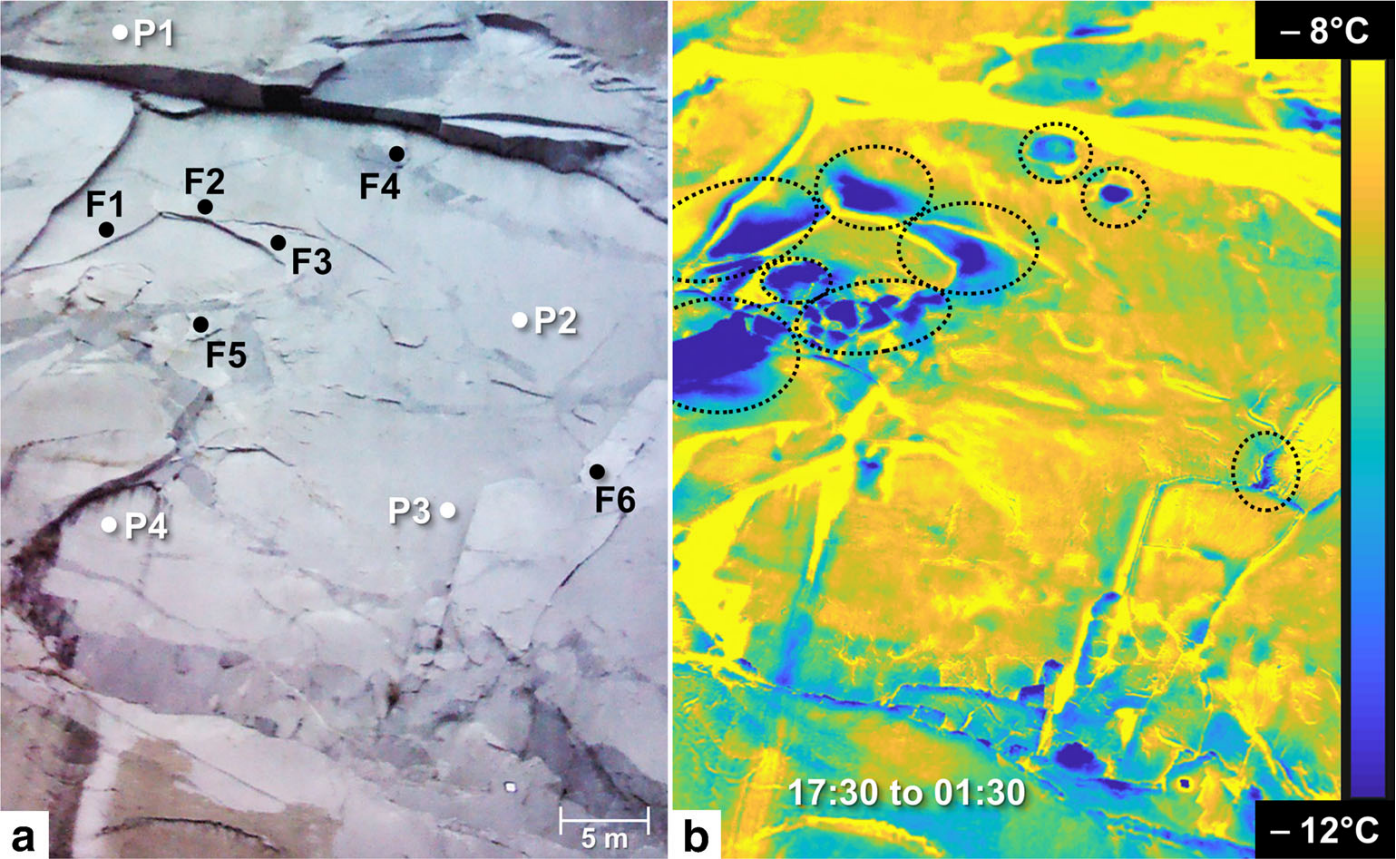
Results of the IRT monitoring conducted on 19–20 Oct. 2015 on El Capitan. (A) Front view of El Capitan study area on 19 Oct. 2015 at 18:10 PDT (stitching of two photos from the thermal camera). F# labels and P# labels correspond to the labelled rock sheets and the reference parts, respectively. Thickness of the rock sheets (values indicated at ± 0.9 mm): F1 = 10.7 cm; F2 = 9.4 cm; F3 = 13.5 cm; F4 = 7.1 cm; F5 = 3.8 cm; and F6 = 7.8 cm. (B) Nocturnal cooling of El Capitan between 17:30 and 01:30 PDT. Dashed black circles indicate the exfoliated areas that have cooled more than the reference areas

**Fig. 9:**
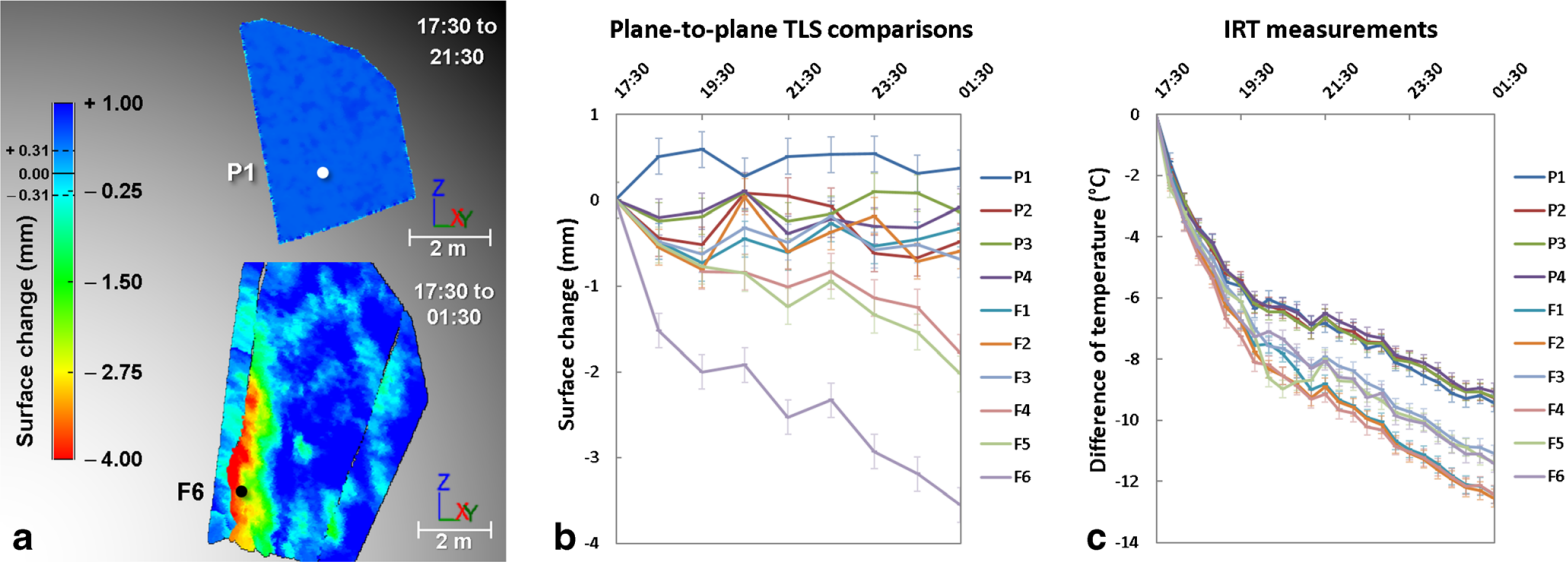
Results of the TLS monitoring conducted on 19–20 Oct. 2015 on El Capitan**.** (A) Filtered point-to-mesh differences measured from the first TLS point cloud acquired on 19 Oct. 2015 at 17:30 PDT, for P1and F6, respectively. Each comparison is characterized by a registration error in the reference areas of ± 0.31 mm (2σ). Negative surface changes indicate an inward deformation pattern that results in a narrowing of the crack width during overnight cooling. Positive surface changes greater than + 0.31 mm are due to border effects (incidence angle errors). (B) Deformations measured in the ten-labelled areas. Only rock sheets “F4,” “F5,” and “F6” are subject to a crack closure movement like the Rhombus Wall flake. Error bars reflect the standard error of the mean of surface changes detected from TLS data. (C) Nocturnal rock cooling measured in the ten-labelled areas. As observed for the Rhombus Wall flake, the six labelled rock sheets have cooled more than the reference areas. Error bars reflect the standard error of the mean of temperature differences measured with the IRT camera

The thermo-deformation characteristics outlined for a “typical” exfoliation sheet (such as we assume is the case for the Rhombus Wall flake) can be exploited for potentially identifying other similar partially detached and deforming exfoliation sheets. To test this approach, we applied high-frequency TLS and IRT surveying to the El Capitan study area. The comparison of the thermograms acquired on this rock wall identified 11 exfoliation sheets (whose the thicknesses are between 3.8 and 13.5 cm; Fig. 8) that behave thermally like the Rhombus Wall flake; their edges are colder on all the thermograms, and their central part undergoes the most significant nocturnal cooling (e.g., Δ_IRT_ of −13.4 ± 1.0 °C for flake “F3” between 17:30 and 01:30 PDT) (Figs. 8B and 9C). These results mirror those observed for the Rhombus Wall flake and allow extrapolating those results because the length of the rock sheets at the El Capitan study area varies from a few decimeters to several meters (Figs. 2C and 8A).

Given these results, we surmise that whatever the rock sheet size, the nocturnal thermal signature of exfoliations sheets is likely to be similar. This demonstrates that IRT offers a means of remote detection of these potential rockfall sources. However, our data also show that not all detected rock sheets are prone to deformation. Of the 11 IRT-detected exfoliation sheets at El Capitan, only three of them underwent a phase of progressive contractions (e.g., see the deformation pattern for flake “F6” in Fig. 9). For these three flakes, however, deformation amplitudes reach −2 to −3 mm over the 8-h monitoring period (Fig. 9B). In contrast, the deformation range associated with the other rock sheets is similar to that observed in the reference areas (e.g., see the deformation pattern for area “P1” in Fig. 9A). For these, overall deformations vary between ± 0.55 mm (Fig. 9B). The reason why only some thermally detected exfoliation sheets show significant deformation may be due to the limits of our TLS detection methods. That is, sheets that undergo small thermal variations may also be undergoing progressive contractions (during cooling phases), but were not detected. However, it is also possible that the lack of progressive deformation is due to the particular geometries of the flake themselves. For example, how and where exfoliation sheets are attached versus detached may very well govern the type of deformations that sheets are capable of undergoing. Notably, our TLS measurements at El Capitan provide some quantification of these potential geometrical influences. The measurements that we performed on the smoothed reference mesh identified that the crack aperture of the deforming rock sheets was greater than 9 cm in all three cases. In addition to having a deep and persistent crack, a large aperture size appears to be the main topographic criterion favoring the remote detection of a thermally induced deformation cycle for exfoliation sheets. Still, it is worth mentioning that the emergence of a thermally induced deformation cycle within a rock sheet is most likely a scale-independent process. Thus, smaller aperture sizes most certainly also generate deformations, but whose amplitudes are too small to be captured with our TLS detection methods. This limitation reinforces the potential of IRT to remotely detect (Figs. 8B and 9C) potentially unstable exfoliation sheets (or which will become unstable in the future) because as demonstrated by Collins and Stock (2016), long-term trends of deformation patterns indicate that crack aperture gradually increases over time, thus favoring the progressive development of increasingly large seasonal and annual deformation cycles.

Exfoliation sheets with large (decimeter-scale) crack apertures were recently investigated using IRT methods on a separate part of El Capitan and showed that the area of rock bridges connecting the sheets to the parent cliff could be identified by their thermal signature (Guerin et al. 2019). That study also detected colder nocturnal thermal signatures with detached parts of exfoliation sheets and further identified that warmer signatures during overall ambient nocturnal cooling were coincident with rock bridges that likely hold the exfoliation sheets in place on the rock cliff. Thus, IRT methods show great promise not only for detecting the locations of exfoliation sheets with large deformation potential, as shown herein, but also for identifying their relative stability. Notably, the study of Guerin et al. (2019) was conducted with IRT instrumentation at a distance greater than 1 km from the wall. This suggests that our detection methods could be applied at much longer distances for truly long-range remote detection of exfoliation sheets.

## Conclusions

We used TLS and IRT methods to characterize the deformation and temperature relations on exfoliated cliffs in Yosemite Valley, where many rockfalls each year are likely triggered by thermally induced deformations. We demonstrated how TLS surveys can image thermally induced millimetric deformations and improve upon data collected by in situ crackmeters by taking advantage of its high spatial density measurements. Coupling TLS and IRT methods showed that the largest deformations of an exfoliated sheet occur where the temperature variations are the highest and crack apertures are the largest, suggesting that IRT alone could be used to remotely identify at least some potential exfoliation-type rockfall source areas subject to thermally induced deformations. Coupled TLS-IRT methods thus provide a new efficient tool to investigate rock mass fatigue, especially that induced by daily and seasonal cyclic thermal deformation (Collins and Stock 2016; Collins et al. 2018).

We note that the Yosemite sites presented here are quite optimal for this kind of investigation, because both rock surface material (and thus thermal emissivity) and orientation are homogenous; as shown by Guerin et al. (2019), other geometric or surface features may cause thermal anomalies on the IRT images (e.g., overhangs or lighter-colored rock areas). Future developments will need to be considered on how to address more complicated cliff lithologies and morphologies for a broader application of TLS-IRT coupling.

## Electronic supplementary material

ESM 1 (DOCX 1536 kb)

ESM 6 (PNG 76 kb)

High Resolution (TIFF 126 kb)

ESM 7 (PNG 120 kb)

High Resolution (TIFF 175 kb)

ESM 8 (XLSX 11 kb)

ESM 9 (XLSX 10 kb)

## Data Availability

The datasets generated during and/or analyzed during the current study are available from the corresponding author on reasonable request.
