# Supplementary material for: Remote thermal detection of exfoliation sheet deformation
Source: Landslides. 2020 Oct 7;18(3):865–79. doi: 10.1007/s10346-020-01524-1 (PMC7936945; doi:10.1007/s10346-020-01524-1)
Supplement: Supplementary file 5 — (DOCX 1536 kb) [file 10346_2020_1524_MOESM1_ESM.docx]

**Remote thermal detection of exfoliation sheet deformation**

**Antoine Guerin, Michel Jaboyedoff, Brian D. Collins, Greg M. Stock, Marc-Henri Derron, Antonio Abellán & Battista Matasci**

**Supplementary Material**

**Supplementary Table 1:** Calibration parameters measured during the IRT monitoring of 13-14 Oct. 2015 on the Rhombus Wall flake.

**Supplementary Table 2:** Calibration parameters measured during the IRT monitoring of 19-20 Oct. 2015 on El Capitan.

**Supplementary Figure 1:** Correlation between deformation values measured with TLS along the Rhombus Wall flake and those given by the crackmeters.

**Supplementary Figure 2:** Correlation between temperatures measured with the thermal camera on the Rhombus Wall flake and those measured by thermoresistance sensors.

**Supplementary Movie 1:** 24 hours of thermally induced deformation highlighted for the Rhombus Wall flake by means of TLS monitoring.

**Supplementary Movie 2:** Cross-sectional view of the daily asymmetric buckling deformation cycle highlighted along the Rhombus Wall flake edge by means of TLS monitoring.

**Supplementary Movie 3:** Cross-sectional view of the daily asymmetric buckling cycle highlighted along the Rhombus Wall flake edge by means of crackmeter measurements.

**Supplementary Movie 4:** 24 hours of infrared thermal signatures highlighted for Rhombus Wall flake by means of IRT monitoring.

**Supplementary Table 1. Calibration parameters measured during the IRT monitoring of 13-14 Oct. 2015 on the Rhombus Wall flake.**

| **Time of thermal image**  (PDT) | **Reflected apparent temperature**  (°C) | **Ambient air temperature**  (°C) | **Relative humidity**  (%) |
| --- | --- | --- | --- |
| 20:00  20:20  20:40 | -26.2  -17.1  -15.4 | 21.8  21.3  20.4 | 32  42  44 |
| 21:00  21:20  21:40 | -17.0  -18.9  -19.7 | 20.2  20.9  20.5 | 44  40  45 |
| 22:00  22:20  22:40 | -20.0  -20.3  -19.2 | 20.2  20.1  19.7 | 44  41  43 |
| 23:00  23:20  23:40 | -20.1  -19.9  -15.8 | 19.9  20.3  20.2 | 45  38  42 |
| 00:00  00:20  00:40 | -18.4  -22.3  -21.9 | 19.6  18.8  18.2 | 42  47  48 |
| 01:00  01:20  01:40 | -22.1  -22.0  -21.7 | 19.1  18.4  18.0 | 43  47  46 |
| 02:00  02:20  02:40 | -21.5  -22.4  -22.2 | 17.7  17.5  17.3 | 45  48  49 |
| 03:00  03:20  03:40 | -21.3  -21.8  -21.4 | 17.8  17.2  16.6 | 45  50  51 |
| 04:00  04:20  04:40 | -21.6  -22.1  -21.7 | 16.6  16.4  16.3 | 51  53  49 |
| 05:00  05:20  05:40 | -22.9  -22.4  -22.3 | 15.9  16.7  16.5 | 53  49  48 |
| 06:00  06:20  06:40 | -21.5  -22.0  -22.8 | 16.4  16.2  15.8 | 53  53  51 |
| 07:00  07:20  07:40 | -22.2  -18.1  -22.7 | 17.6  16.1  16.8 | 44  53  50 |
| 08:00  08:20  08:40 | -23.3  -19.4  -19.1 | 17.4  17.1  16.2 | 47  53  54 |
| 09:00  09:20  09:40 | -21.8  -18.2  -18.8 | 17.1  18.4  19.5 | 54  50  49 |
| 10:00  10:20  10:40 | -20.1  -16.5  -16.3 | 20.8  22.3  22.6 | 44  43  42 |
| 11:00  11:20  11:40 | -15.2  -7.0  -13.9 | 23.3  23.8  24.5 | 34  34  32 |
| 12:00  12:20  12:40 | -11.7  -6.3  -3.1 | 26.4  27.8  26.5 | 30  28  27 |
| 13:00  13:20  13:40 | -8.8  -10.2  -1.4 | 27.0  26.8  26.5 | 29  28  28 |
| 14:00  14:20  14:40 | -2.0  -8.5  +4.9 | 27.4  27.6  26.8 | 29  28  27 |
| 15:00  15:20  15:40 | -7.1  -7.7  -8.6 | 27.3  27.7  27.9 | 29  27  27 |
| 16:00  16:20  16:40 | +0.2  -11.3  -11.2 | 27.9  27.3  26.6 | 25  27  28 |
| 17:00  17:20  17:40 | -11.4  -8.9  -4.8 | 26.3  26.3  25.8 | 28  29  29 |
| 18:00  18:20  18:40 | -5.1  -2.0  -1.7 | 25.6  25.1  24.7 | 29  29  32 |
| 19:00  19:20  19:40 | -5.3  -6.2  -5.6 | 24.4  23.9  23.5 | 31  30  32 |
| 20:00 | -7.9 | 22.8 | 34 |

**Supplementary Table 2. Calibration parameters measured during the IRT monitoring of 19-20 Oct. 2015 on El Capitan.**

| **Time of thermal image**  (PDT) | **Reflected apparent temperature**  (°C) | **Ambient air temperature**  (°C) | **Relative humidity**  (%) |
| --- | --- | --- | --- |
| 17:30  17:50 | -33.7  -28.1 | 14.7  14.2 | 61  61 |
| 18:10  18:30  18:50 | -30.4  -23.5  -26.8 | 14.0  13.7  13.1 | 60  60  62 |
| 19:10  19:30  19:50 | -29.0  -29.2  -24.6 | 12.8  12.3  11.8 | 66  69  70 |
| 20:10  20:30  20:50 | -22.9  -24.3  -23.8 | 12.3  12.2  12.0 | 69  70  71 |
| 21:10  21:30  21:50 | -26.4  -32.1  -29.9 | 11.7  11.3  10.9 | 71  75  76 |
| 22:10  22:30  22:50 | -29.7  -32.2  -31.0 | 11.2  11.1  10.9 | 77  75  76 |
| 23:10  23:30  23:50 | -31.9  -32.5  -33.8 | 10.6  10.5  10.2 | 81  81  79 |
| 00:10  00:30  00:50 | -34.1  -33.3  -33.4 | 10.1  9.8  9.6 | 79  79  80 |
| 01:10  01:30 | -33.2  -33.7 | 10.1  9.2 | 70  82 |


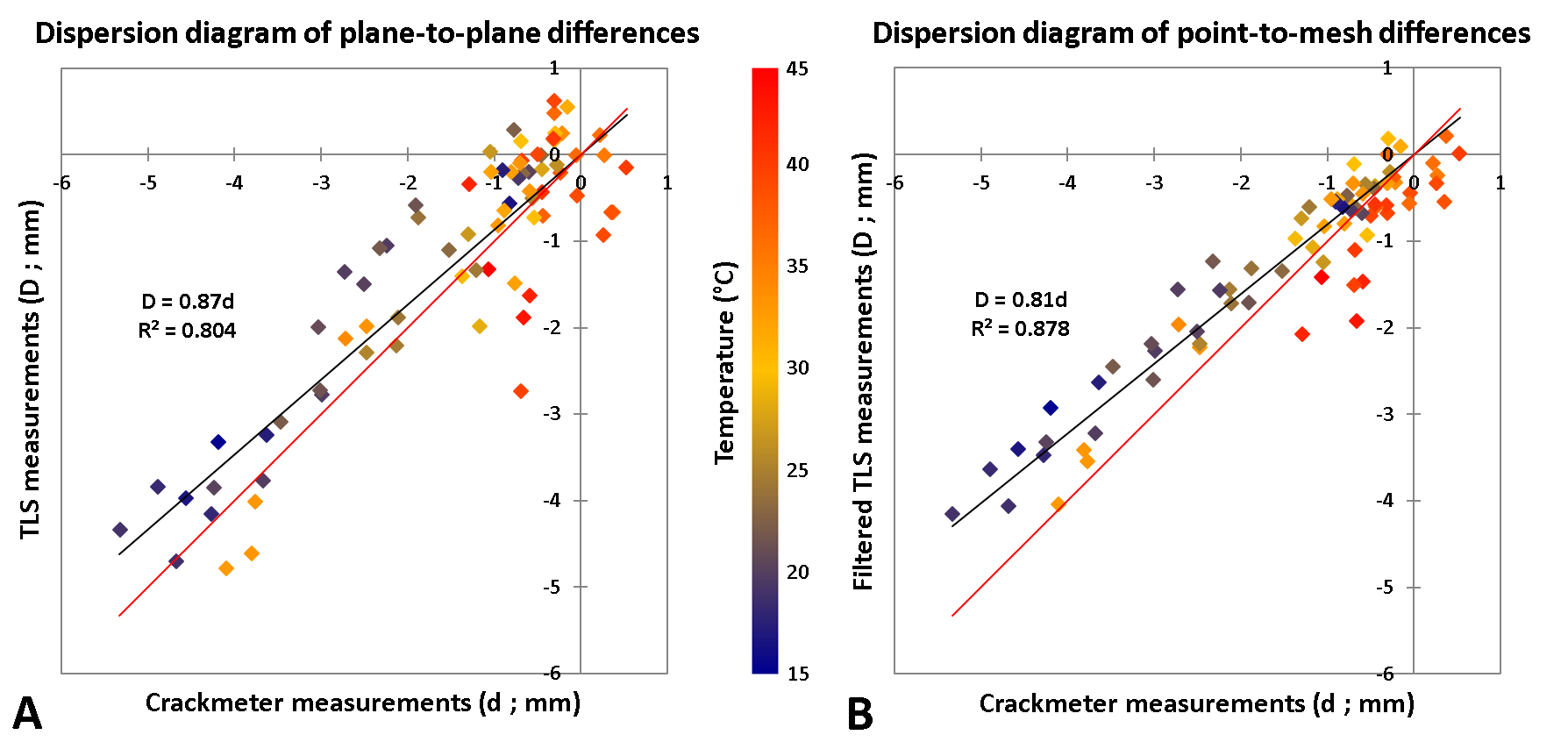
 **Supplementary Figure 1. Correlation between deformation values measured with TLS along the Rhombus Wall flake and those given by the crackmeters.** Given there is twice as much TLS data (acquired every hour) as crackmeter data (recorded every two hours), only TLS data acquired at even hours were used in these scatter plots. **(A)** Dispersion diagram (all crackmeter combined) of plane-to-plane TLS distances (Fig. 5B) versus crackmeter measurements (Fig. 5C). Data points were colored according to their temperature measured with the IRT. Red line indicates the correlation for R^2^ = 1. **(B)** Dispersion diagram (all crackmeter combined) of filtered point-to-mesh TLS distances (Fig. 5D) versus crackmeter measurements (Fig. 5C). Data points were colored according to their temperature measured with the IRT. Red line indicates the correlation for R^2^ = 1. Due to the sliding average method, the filtered point-to-mesh distances globally underestimate the deformation values (Figs 3B and 5D).


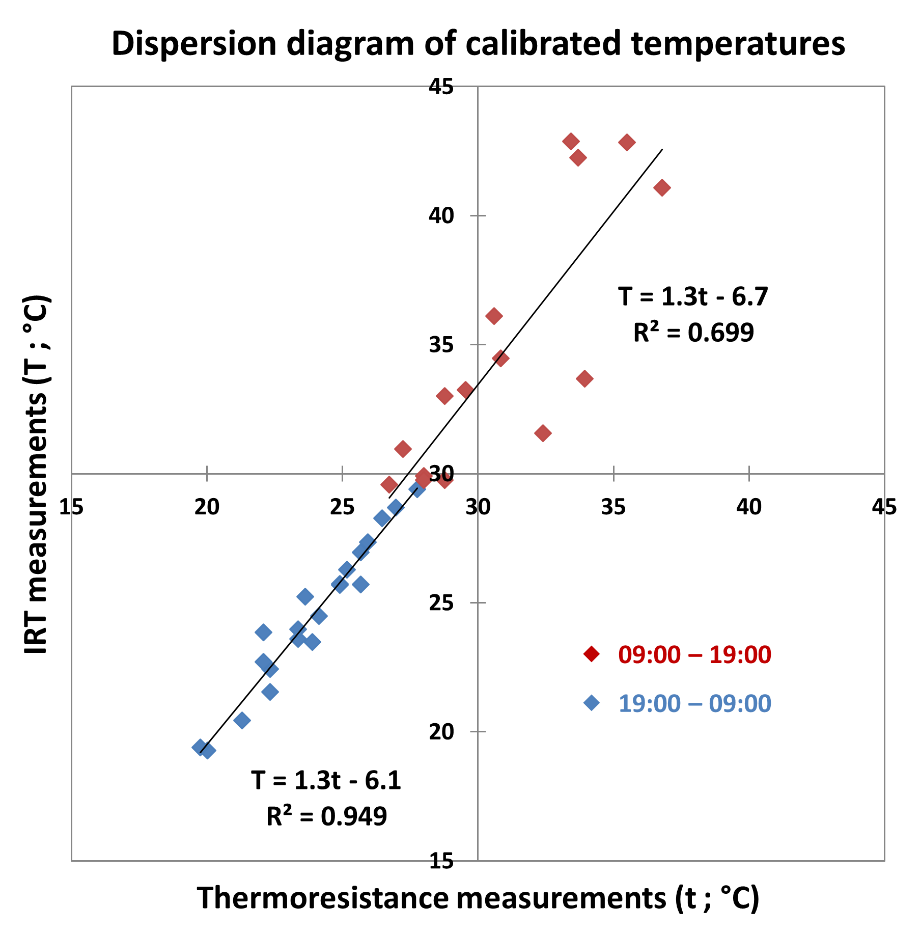


**Supplementary Figure 2. Correlation between temperatures measured with the thermal camera on the Rhombus Wall flake and those measured by thermoresistance sensors.** Given that there is six times more IRT data (acquired every 20 minutes) as thermoresistance sensor data (recorded every two hours), only the IRT data acquired at even hours were used in this scatter plot. For all of the thermoresistance data combined, the dispersion diagram indicates that nocturnal calibrated temperatures (from 19:00 to 09:00 PDT) are very close (although overall overestimated) to the thermoresistance data (average deviation in absolute value: 0.9°C). Due to direct and indirect solar radiation, temperature overestimation is higher with diurnal calibrated temperatures (from 09:00 to 19:00 PDT). The average deviation in absolute value with the thermoresistance data is 3.9°C.


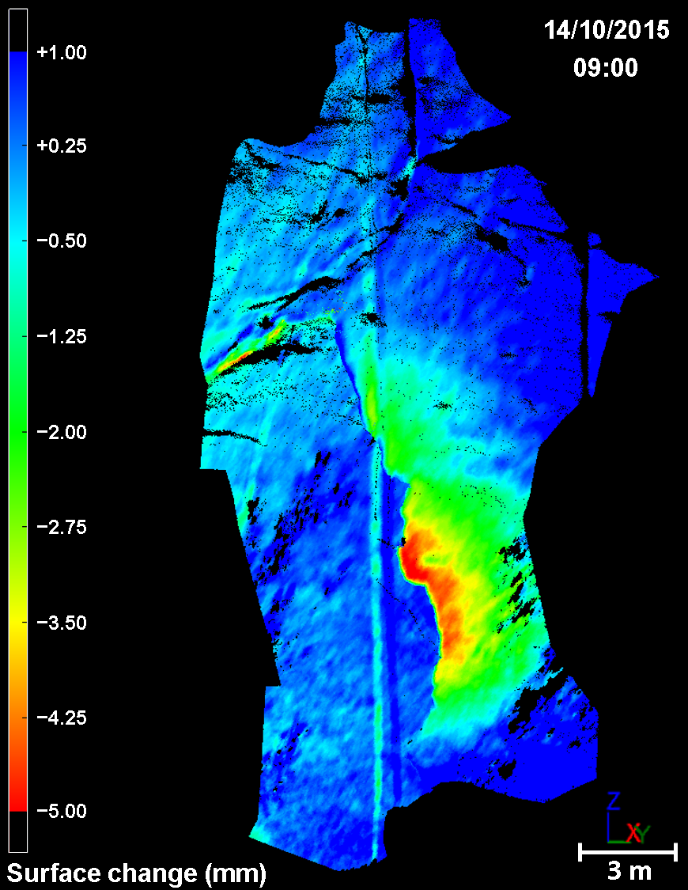


**Supplementary Movie 1. 24 hours of thermally induced deformation highlighted for the Rhombus Wall flake by means of TLS monitoring.** The video shows the cumulative hourly evolution of the filtered point-to-mesh differences measured from the first point cloud acquired on 13 Oct. 2015 at 20:00 PDT. Each comparison is characterized by a change detection uncertainty of ± 0.42 mm. Negative surface changes indicate an inward deformation pattern that results in a narrowing of the crack width during overnight cooling. Positive surface changes greater than + 0.42 mm (dark blue color) are due to border effects (incidence angle errors).


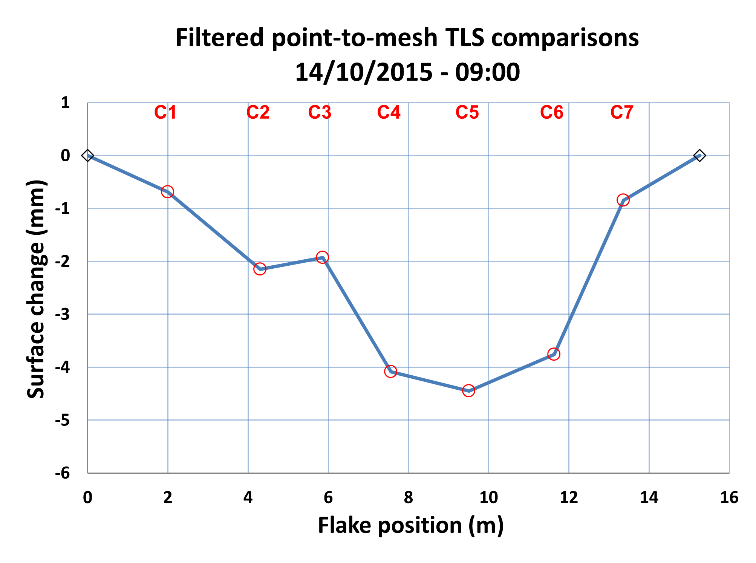


**Supplementary Movie 2. Cross-sectional view of the daily asymmetric buckling deformation cycle highlighted along the Rhombus Wall flake edge by means of TLS monitoring.** Key to abbreviation: C# = Crackmeter. The video shows the cumulative hourly evolution of the filtered point-to-mesh differences measured at the position of each crackmeter from the first point cloud acquired on 13 Oct. 2015 at 20:00 PDT. Buckling characterization: First mode: 20:00 – 12:00 PDT then 17:00 – 20:00 PDT; Second mode: 12:00 – 17:00 PDT.


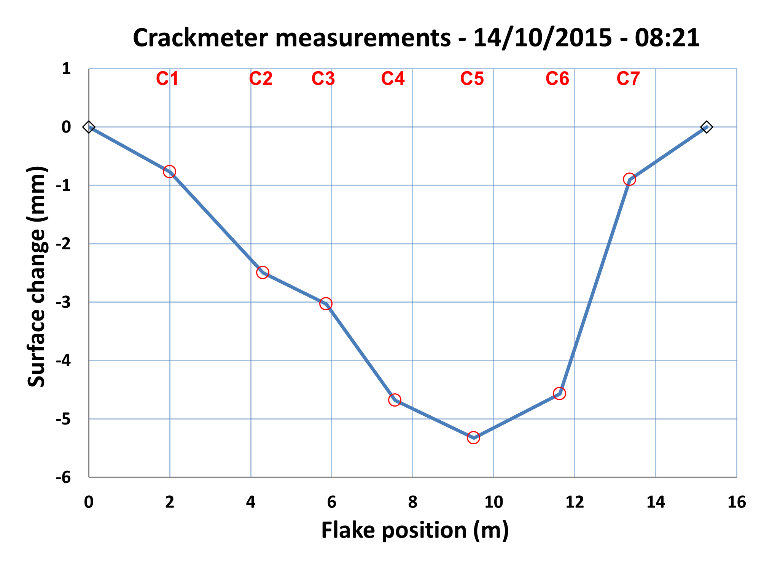


**Supplementary Movie 3. Cross-sectional view of the daily asymmetric buckling cycle highlighted along the Rhombus Wall flake edge by means of crackmeter measurements.** The video shows the cumulative evolution at 2-hour interval of the deformations recorded by the seven crackmeters (C1 through C7) from the first reading performed on 13 Oct. 2015 at 20:24 PDT. Buckling characterization: First mode: 20:00 – 12:00 PDT then 17:00 – 20:00 PDT; Second mode: 12:00 – 17:00 PDT.


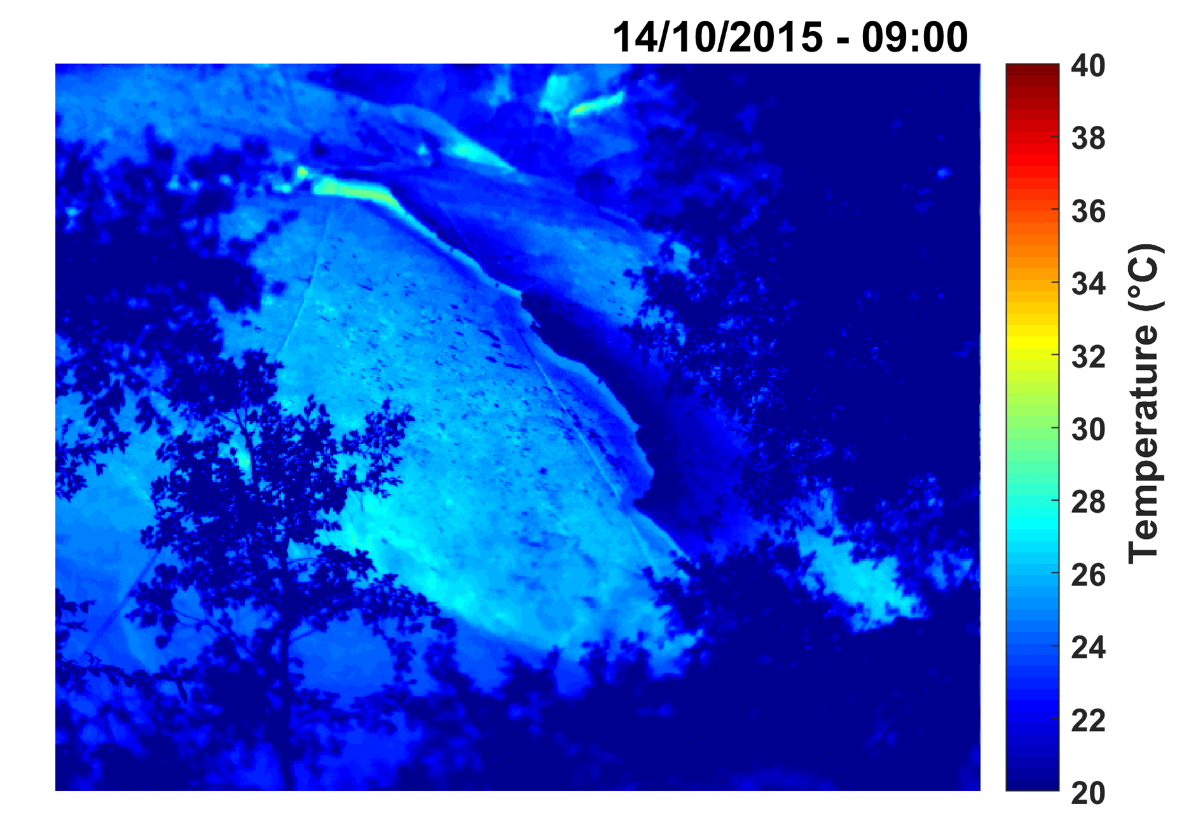


**Supplementary Movie 4. 24 hours of infrared thermal signatures highlighted for Rhombus Wall flake by means of IRT monitoring.** The video shows the evolution of surface temperatures at 20-minute interval between 13 Oct. 2015 at 20:00 PDT and 14 Oct. 2015 at 20:00 PDT. The temperature scale is the same for all thermograms.
