# Supplementary figures and images for: Remote thermal detection of exfoliation sheet deformation
Source: Landslides. 2020 Oct 7;18(3):865–79. doi: 10.1007/s10346-020-01524-1 (PMC7936945; doi:10.1007/s10346-020-01524-1)

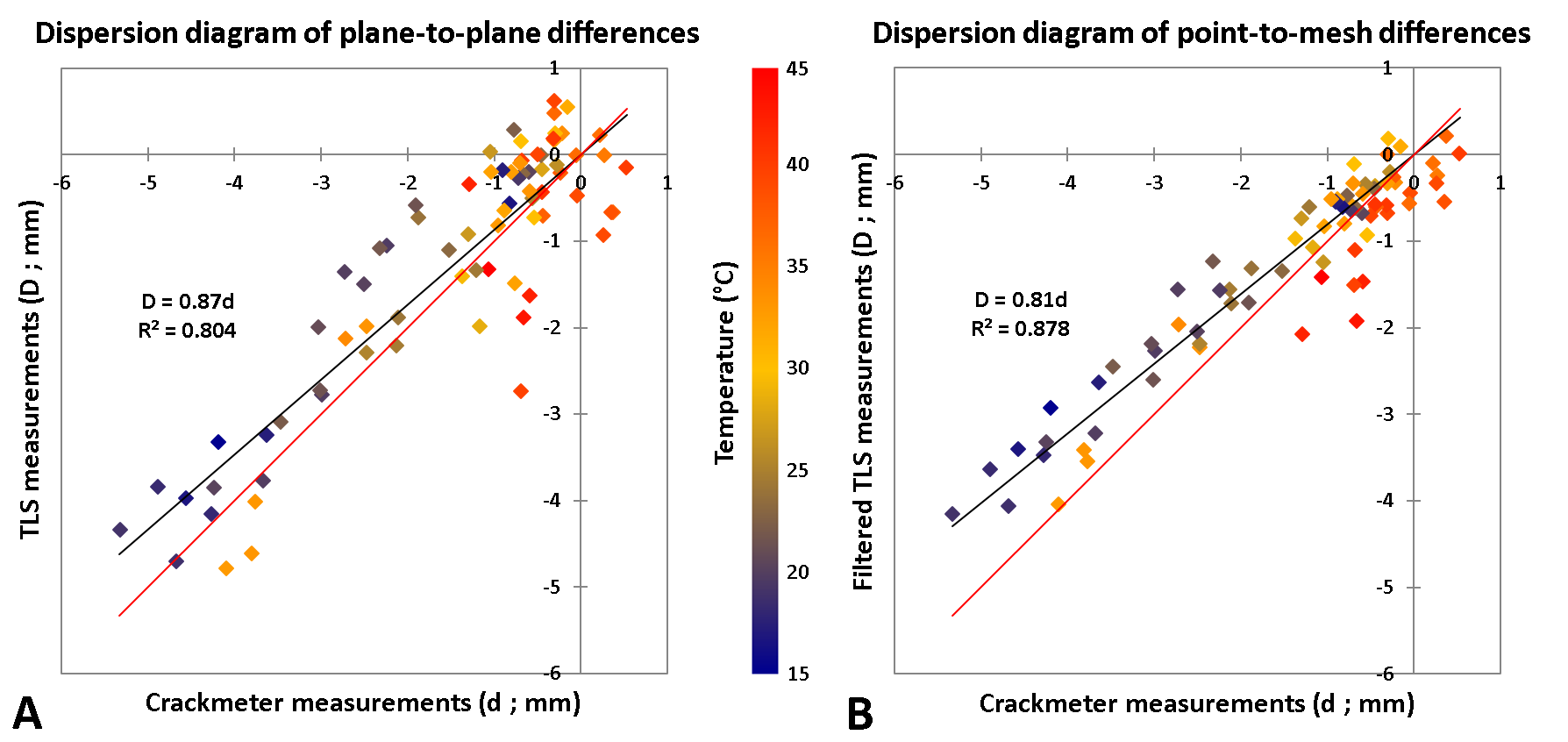

Supplement: Supplementary file 6 — (PNG 76 kb) [file 10346_2020_1524_Fig10_ESM.png]

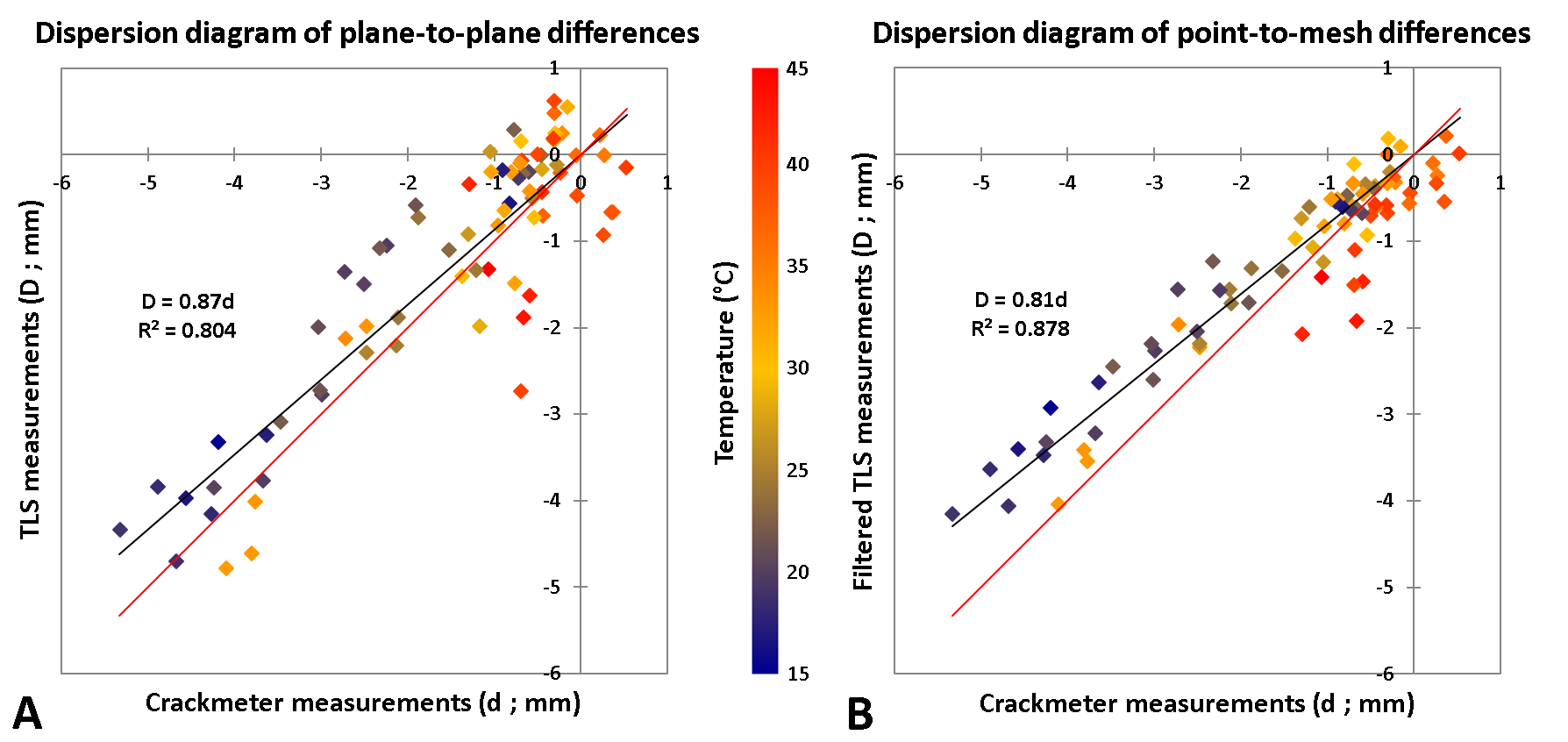

Supplement: Supplementary file 7 — High Resolution (TIFF 126 kb) [file 10346_2020_1524_MOESM6_ESM.tiff]

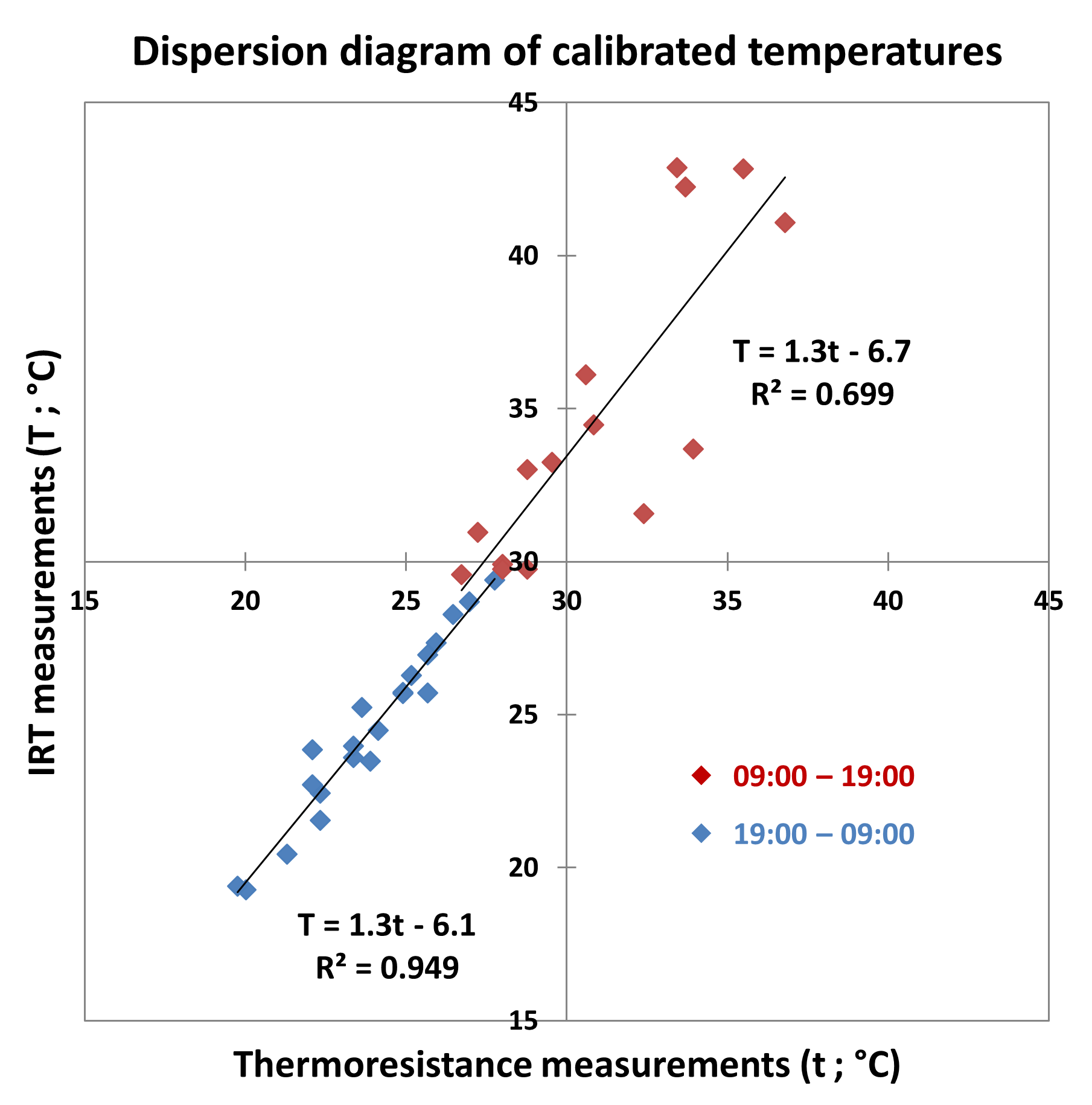

Supplement: Supplementary file 8 — (PNG 120 kb) [file 10346_2020_1524_Fig11_ESM.png]

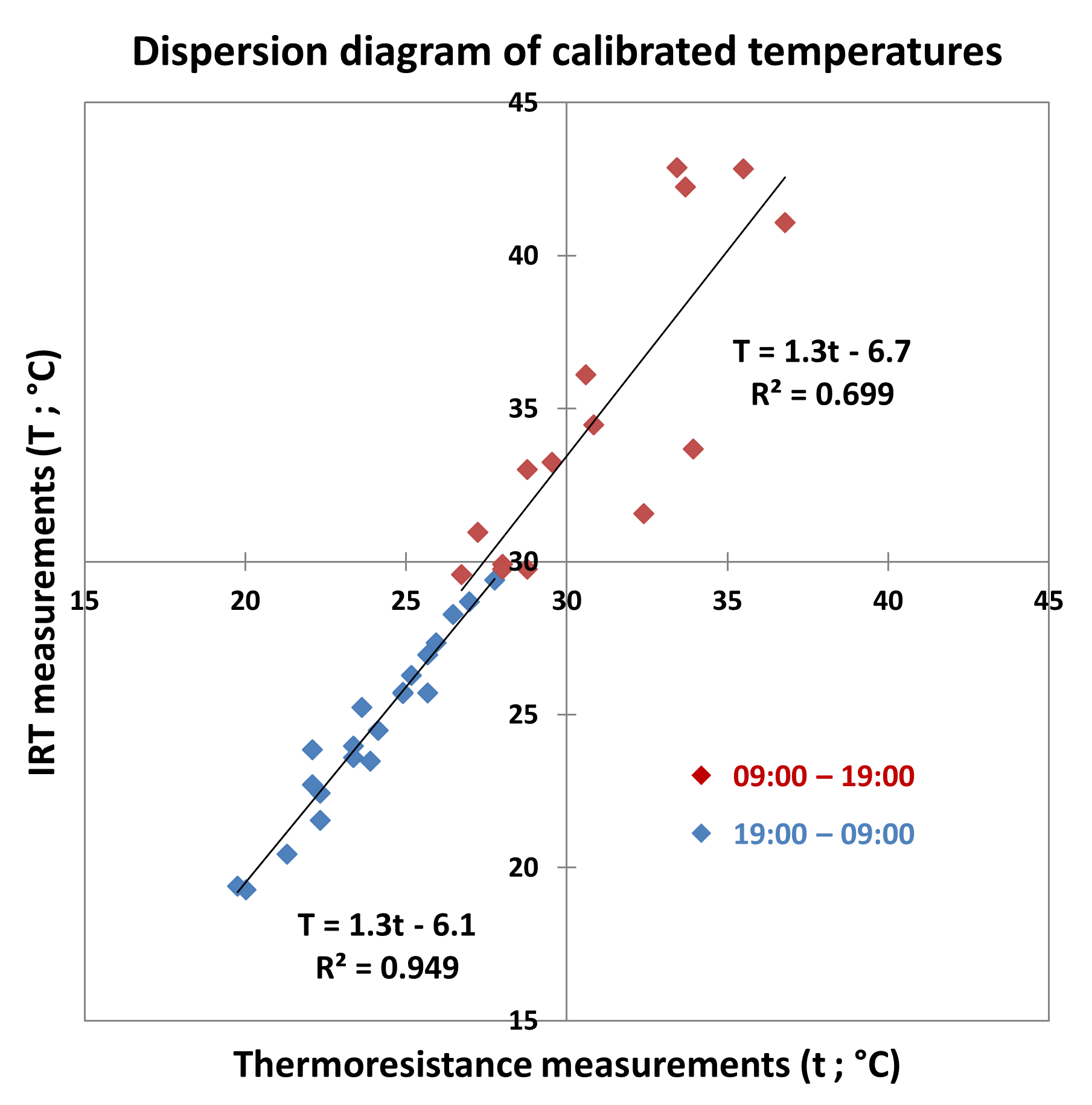

Supplement: Supplementary file 9 — High Resolution (TIFF 175 kb) [file 10346_2020_1524_MOESM7_ESM.tiff]
